# Neurological manifestations of SARS-CoV-2: complexity, mechanism and associated disorders

**DOI:** 10.1186/s40001-023-01293-2

**Published:** 2023-08-30

**Authors:** Kritika Tyagi, Prachi Rai, Anuj Gautam, Harjeet Kaur, Sumeet Kapoor, Ashish Suttee, Pradeep Kumar Jaiswal, Akanksha Sharma, Gurpal Singh, Ravi Pratap Barnwal

**Affiliations:** 1 Department of Biophysics, Panjab University, Chandigarh, India; 2 Centre for Biomedical Engineering, Indian Institute of Technology, New Delhi, India; 3 School of Pharmaceutical Sciences, Lovely Professional University, Phagwara, India; 4 Department of Biochemistry and Biophysics, Texas A & M University, College Station, TX 77843 USA; 5 University Institute of Pharmaceutical Sciences, Panjab University, Chandigarh, India

**Keywords:** SARS-CoV-2, COVID, Blood–brain barrier, Central nervous system, Neuroinvasion, ACE-2, Cerebrovascular disease

## Abstract

**Background:**

Coronaviruses such as Severe Acute Respiratory Syndrome coronavirus (SARS), Middle Eastern Respiratory Syndrome (MERS) and Severe Acute Respiratory Syndrome Coronavirus 2 (SARS-CoV-2) are associated with critical illnesses, including severe respiratory disorders. SARS-CoV-2 is the causative agent of the deadly COVID-19 illness, which has spread globally as a pandemic. SARS-CoV-2 may enter the human body through olfactory lobes and interact with the angiotensin-converting enzyme2 (ACE2) receptor, further facilitating cell binding and entry into the cells. Reports have shown that the virus can pass through the blood–brain barrier (BBB) and enter the central nervous system (CNS), resulting in various disorders. Cell entry by SARS-CoV-2 largely relies on TMPRSS2 and cathepsin L, which activate S protein. TMPRSS2 is found on the cell surface of respiratory, gastrointestinal and urogenital epithelium, while cathepsin-L is a part of endosomes.

**Aim:**

The current review aims to provide information on how SARS-CoV-2 infection affects brain function.. Furthermore, CNS disorders associated with SARS-CoV-2 infection, including ischemic stroke, cerebral venous thrombosis, Guillain–Barré syndrome, multiple sclerosis, meningitis, and encephalitis, are discussed. The many probable mechanisms and paths involved in developing cerebrovascular problems in COVID patients are thoroughly detailed.

**Main body:**

There have been reports that the SARS-CoV-2 virus can cross the blood–brain barrier (BBB) and enter the central nervous system (CNS), where it could cause a various illnesses. Patients suffering from COVID-19 experience a range of neurological complications, including sleep disorders, viral encephalitis, headaches, dysgeusia, and cognitive impairment. The presence of SARS-CoV-2 in the cerebrospinal fluid (CSF) of COVID-19 patients has been reported. Health experts also reported its presence in cortical neurons and human brain organoids. The possible mechanism of virus infiltration into the brain can be neurotropic, direct infiltration and cytokine storm-based pathways. The olfactory lobes could also be the primary pathway for the entrance of SARS-CoV-2 into the brain.

**Conclusions:**

SARS-CoV-2 can lead to neurological complications, such as cerebrovascular manifestations, motor movement complications, and cognitive decline. COVID-19 infection can result in cerebrovascular symptoms and diseases, such as strokes and thrombosis. The virus can affect the neural system, disrupt cognitive function and cause neurological disorders. To combat the epidemic, it is crucial to repurpose drugs currently in use quickly and develop novel therapeutics.

## Highlights

- SARS-CoV-2, causative agent of COVID-19 which halted the world, has neurological implications.
- Viruses infiltrate the nervous system by vesicle-mediated transport, transcytosis, molecular envelope technology, neurotropism, and direct invasion.
- Olfactory invasion by SARS-CoV-2 and ACE2 expression in the brain lead to an increase in lung infection cases.
- Encephalitis, Multiple Sclerosis, Ischemic Stroke etc. are some of the diseases which are a result of neuroinvasion by SARS-CoV-2.

## Introduction

The coronavirus disease 2019 (COVID-19) is caused by the Severe Acute Respiratory Syndrome Coronavirus 2 (SARS-CoV-2). It began in Wuhan, China and spread worldwide, resulting in a pandemic [1]. In the past, two more coronavirus outbreaks, the severe acute respiratory syndrome (SARS-CoV) epidemic (2003) and the Middle East Respiratory Syndrome (MERS-CoV) (2012), have led to severe infections and fatalities [2]. Droplets from the infected person's cough, sneeze, and breath serve as the first source of the infection. Infection symptoms appear within 2–14 days of the virus reaching the throat through the mucous membrane [3]. Lungs are the primary target, and hence, regular breathing is impaired. COVID-19 cause respiratory tract infections with symptoms, such as fever, cough, and shortness of breath. Symptoms can range from mild, such as a cold or pneumonia, to acute and severe respiratory insufficiency [4]. The first whole genome of SARS-CoV-2, a novel coronavirus similar to SARS-CoV, was recovered from the extracellular fluid of a patient in January 2020 [5]. The genome size of the pathogen varies from 29.8 to 29.9 kb [6]. SARS-CoV-2 is a beta type RNA virus having positive-sense with a single-stranded genome, having 89.1% sequence identity to a group of coronaviruses similar to SARS. SARS-CoV-2 is among the species of the coronaviruses family, which usually causes infections in humans and belongs to the genus Betacoronavirus and sub-genus Sarbecovirus. Four of these human coronaviruses, NL63, KU1, 229E, and OC43, induce common cold symptoms, while the other three are responsible for a variety of fatal infections, that consists of SARS (2002 and 2003) and MERS (2012) [3, 4]. Four coronaviruses cause mild cold-like symptoms, while SARS and MERS have led to severe and fatal infections. Computational techniques are used to analyse the possible influence of critical mutations on SARS-CoV-2 structural proteins and identify important mutations in SARS-CoV-2 genomes [6–8].

SARS-CoV-2 can affect the brain by invading the neural system, causing physiological and psychological effects. It enters through the bloodstream or peripheral nerves, taking over cellular processes and leading to the loss of neuronal function without causing neuron death [9–11]. Neurological symptoms are more prevalent in patients with severe infections than those with milder infections. Viral encephalitis due to COVID-19, leading to neuro infection has been reported for a patient in Beijing [12]. Upon genome sequencing, the researchers stated that SARS-CoV-2 RNA was found in the cerebral fluid, indicating that the virus has the potential to be neuroinvasive [9–13]. It is unclear how SARS-CoV-2 infection causes neurologic symptoms and how SARS-CoV-2 enters the CNS. It is evident from various studies that mild neuropathological alterations are observed in the brain, with brainstem inflammation being the most frequently noted change. Research on SARS-CoV-2's impact on the nervous system is ongoing [14–17]. Brainstem inflammation is a common finding, but the cause of neurological symptoms and how the virus enters the CNS is unclear. Two theories suggest that the virus may enter the CNS through neurotropism or direct infiltration. It is essential to study neuropathological symptoms and compare them to pre-existing conditions and invasive therapies used in severe COVID-19 cases [18].

Due to increasing evidence of COVID-19 infection impacting the brain and neural functioning, there is a growing interest in studying the impact of the virus on the brain. This article focuses on the neurological aspects of COVID-19 and how the virus enters the brain. In addition, neurological diseases linked to the crossing of the blood–brain barrier (BBB), hindering the brain's normal functioning, by the virus are also discussed. Besides, the review provides an overview of the possible routes for entry for the viruses, severe disorders brought on by their long-term effects on the brain, and disease pathways. Furthermore, the risk factors for COVID-19-induced illnesses are also discussed. Studies have revealed that the virus can penetrate the BBB and reach the CNS, where it can cause a variety of life-threatening infections, physiological abnormalities, and blood clots [19]. Finally, the review highlights key pathways (respiratory failure, glucose fluctuations, and psychological stress) which may lead to nervous system diseases such as Guillain–Barre syndrome (GBS), multiple sclerosis, meningitis, and encephalitis, as well as cerebrovascular diseases, such as ischemic stroke and cerebral venous thrombosis. Furthermore, epidemiological studies are needed to monitor long-term health effects and better analyse the risk variables linked to severe complications in COVID-19 patients. There is growing concern about COVID-19's impact on the brain. The virus can enter the brain, leading to life-threatening infections, physiological abnormalities, and blood clots. Long-term effects and risk factors are still being studied. This review summarizes the virus's neuroinvasion and related diseases.

## Blood–brain barrier (BBB)

**Fig. 1:**
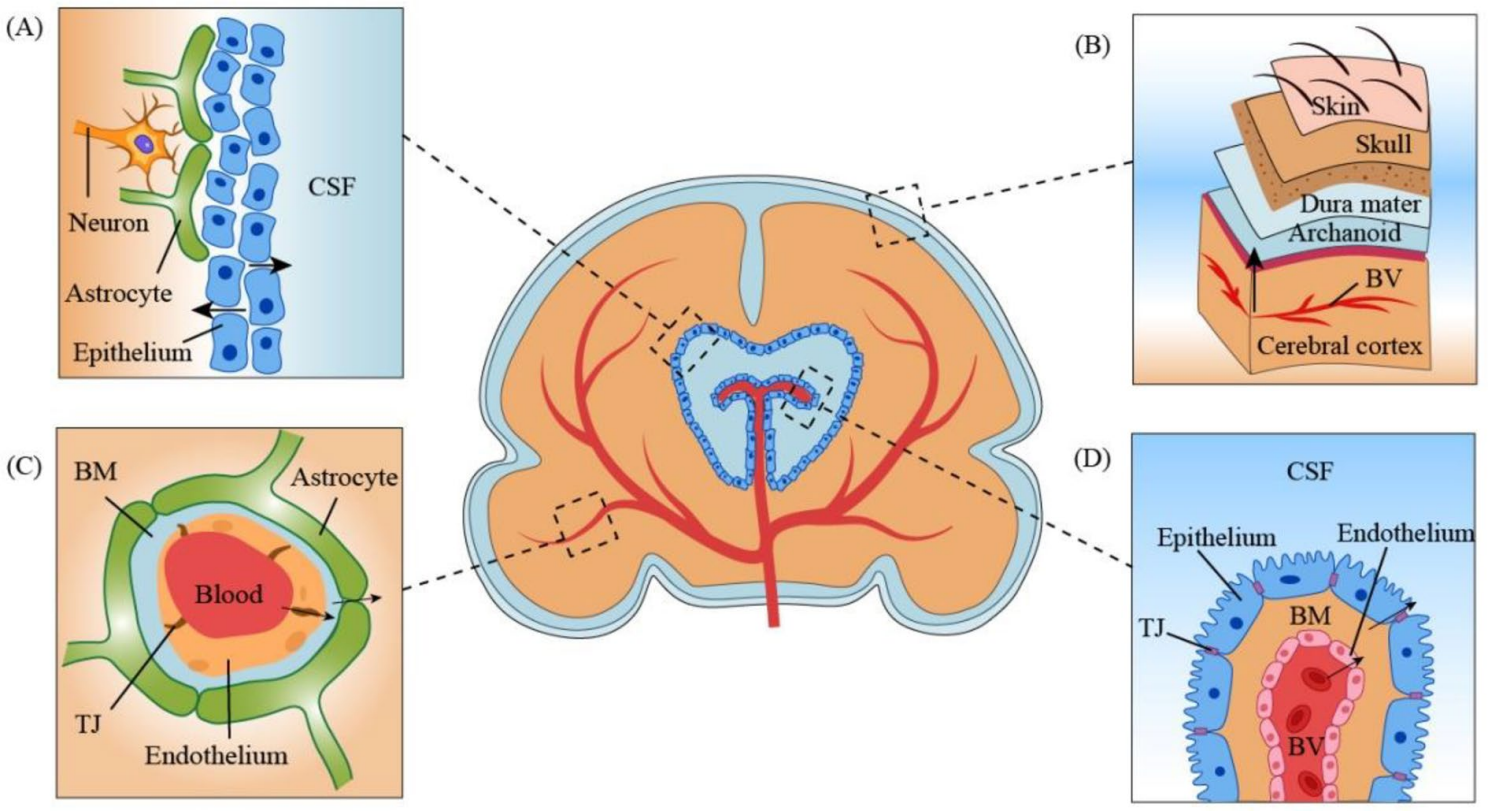
Diagram of different barriers of the brain (potential pathways for neuroinvasion). **A** Brain–CSF barrier. Neuroinvasion from CSF to the cerebral cortex through epithelial cells (free flow in the adult brain) [24]. **B** Blood–brain–meningeal barrier. Meningeal invasion of the virus from BV to various layers(may lead to viral meningitis) [25]. **C** Blood–brain barrier. Potential BBB (capillary endothelial cells with TJ)-mediated invasion of SARS-CoV-2 in the central nervous system [24]. **D** Blood–CSF barrier. Potential blood–CSF barrier-mediated SARS-CoV-2 neuroinfiltration in the central nervous system(through the BM and ependymal cells) [26]. (BV—blood vessel; TJ—tight junctions; BM—basement membrane; CSF—the cerebrospinal fluid)

The brain contains four different kinds of barriers, as shown in Fig. 1. One of these is the membrane separating the blood from the cerebrospinal fluid (CSF), known as the blood–CSF barrier, while the other is the blood–brain barrier (BBB), separating the blood from the fluid inside the brain [20]. The BBB serves as a conduit between blood and various regions in the brain, and it regulates the exchange of various molecules between the blood and the brain. It is a highly selective, protective interface of the brain which permits the selective and active transport of different ions, nutrients, organic anions, and macromolecules like amino acids and glucose essential for brain functioning and prevents the entry of intoxicants and the potential neurotransmitters not suitable for neural function. It also averts the entry of cells, microbial pathogens, and neurotoxic debris into the brain. A healthy BBB provides effective but incomplete protection of the central nervous system (CNS) [21]. Because of its permeability, the CNS is susceptible to invasion by peripheral cells and other microbes, raising questions regarding its defence. One of the leading causes of deaths worldwide is pathogens that can cross the BBB. These include *Escherichia coli* K1, West Nile virus, *Neisseria meningitidis *, *Treponema palladium*, and Human immunodeficiency virus [22]. Macromolecules such as thrombin, albumin, plasminogen, and plasmin are regulated for passage across the BBB, because they tend to damage nerve tissues and interstitial fluid and generation of a cascade cause seizures, cell death, glial cell division, among other things [23].

### Structure of the blood–brain barrier

**Fig. 2:**
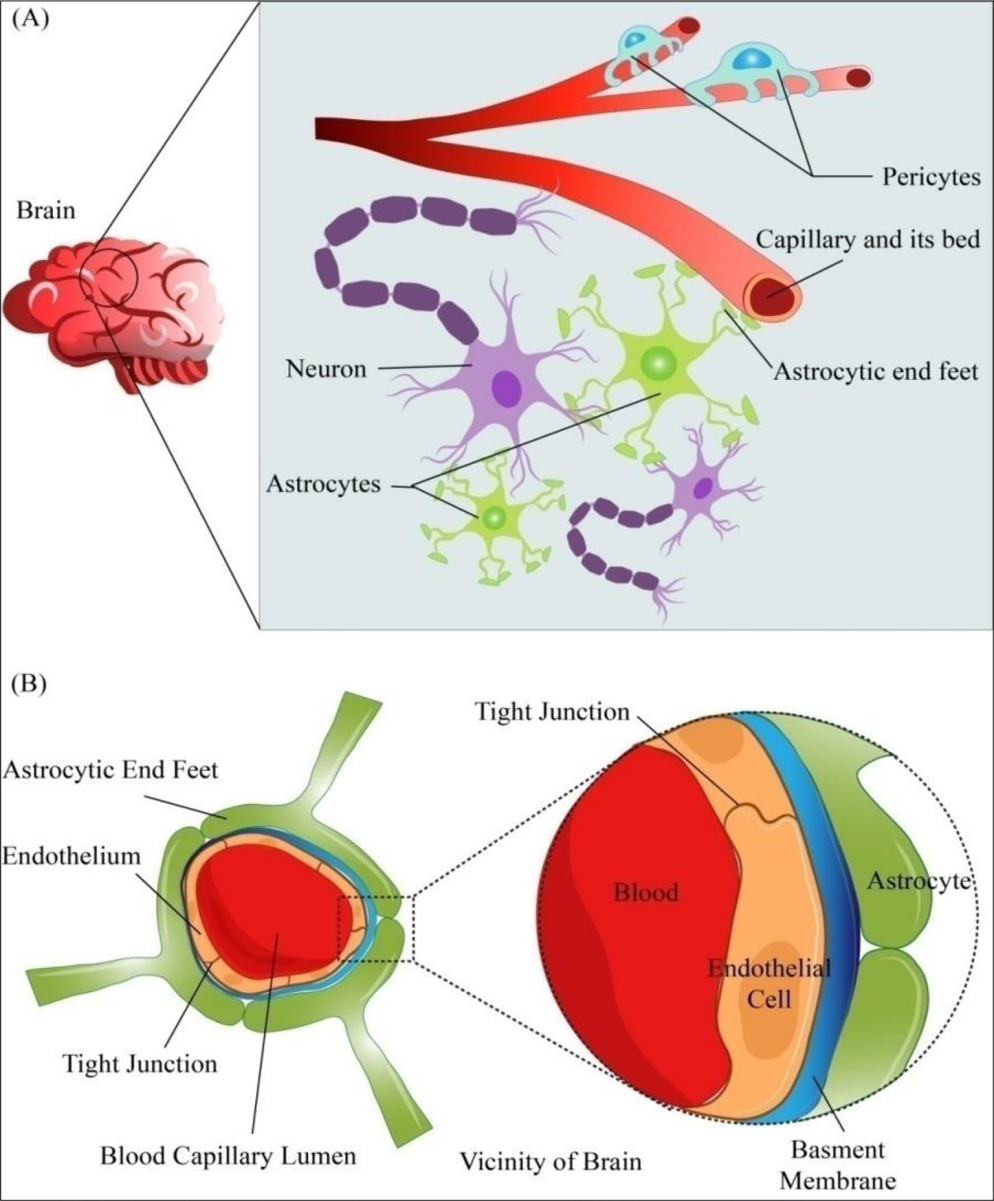
**A** Endothelium is covered by pericytes and astrocytic end-feet, which secrete the matrix proteins that make up the basement membrane. Neurons, pericytes, and astrocytes surrounding the blood capillary are shown. Astrocytes are stacked around the brain's blood arteries and are considered to be in charge of moving ions out of the brain. Due to their integration into the basement membrane micro vessels, pericytes strongly connect with the endothelial cells that make up the BBB. They provide structural integrity to the micro vessels. **B** Cross-sectional view of the blood vessel and schematic representation of layers present in the blood–brain barrier shows the outermost astrocytes separated from endothelial cells and tight junctions within these cells by a basement membrane [30]

The human body contains three different types of capillary networks. These three types of capillaries are sinusoidal, fenestrated, and continuous. The endothelial cell-based lining of the body consists of continuous capillaries. Since these cells line up one after the other, they are called continuous. The large openings between the cells allow fast substance exchange in continuous, fenestrated capillaries [25]. Kidneys, small intestine, and endocrine glands comprise these types of capillaries. The continuous capillaries are further of two types: continuous-fenestrated and continuous non-fenestrated. Only small molecules such as water, glucose, hormones, and gases can pass through the gaps in the lining of continuous non-fenestrated capillaries known as intracellular clefts [24]. These capillaries are in the nervous system, skin, and lungs. Sinusoidal capillaries, also known as discontinuous capillaries, have bigger holes and gaps. Sinusoidal capillaries are present in the bone marrow, lymph nodes, endocrine glands, liver and spleen. The discontinuous capillaries are the most absorbent, while the continuous ones are the least permeable. Fenestrated capillaries have pores in their endothelial cells to facilitate more movement than continuous capillaries. Since the capillaries that BBB uses are unique, they are comprised of continuous, non-fenestrated capillaries with strong connections between their endothelial cells, which prevents paracellular trafficking [27]. The basic structure of the BBB is depicted in Fig. 2. These cells can precisely control the flow of infections and undesirable substances between the blood and the brain due to the absence of gaps between the endothelial cells in these capillaries, and they also lack pinocytic vacuoles. These endothelial cells also have adherent junctions and tight junctions. Only molecules with specific mass and lipophilicity can pass through the CNS's endothelial cells because of their high levels of selectiveness. The BBB is further supported structurally by the tight and adherent junctions that hold endothelial cells tightly together at the interface. The primary purpose of these tight junctions is to stop ions, large macromolecules and other soluble compounds from passing through them via paracellular diffusional channels [28]. Occludin, claudins, and other proteins that traverse the intercellular cleft make up tight junctions. These proteins are connected to crucial scaffolding proteins and other regulatory proteins, including Zona Occludens (ZO)-1, 2, 3, and Cingulin. Among all the isoforms of claudins reported to date, in vivo research has shown that claudin-5 and claudin-3 are necessary for proper BBB function [28]. The basal lamina is the second line of defence, which comprises collagen, heparin sulphate, proteoglycans and laminin. Besides this, the basal lamina houses pericytes and macrophages responsible for forming tight junctions and reducing vascular permeability. The glial cells or the astrocytic end feet are the third lines of defence along with angiotensin and glial cells derived neurotrophic factors, which maintain the ionic balance, and aid in the repair mechanism of the BBB [19, 21].

### Transport across the blood–brain barrier

**Fig. 3:**
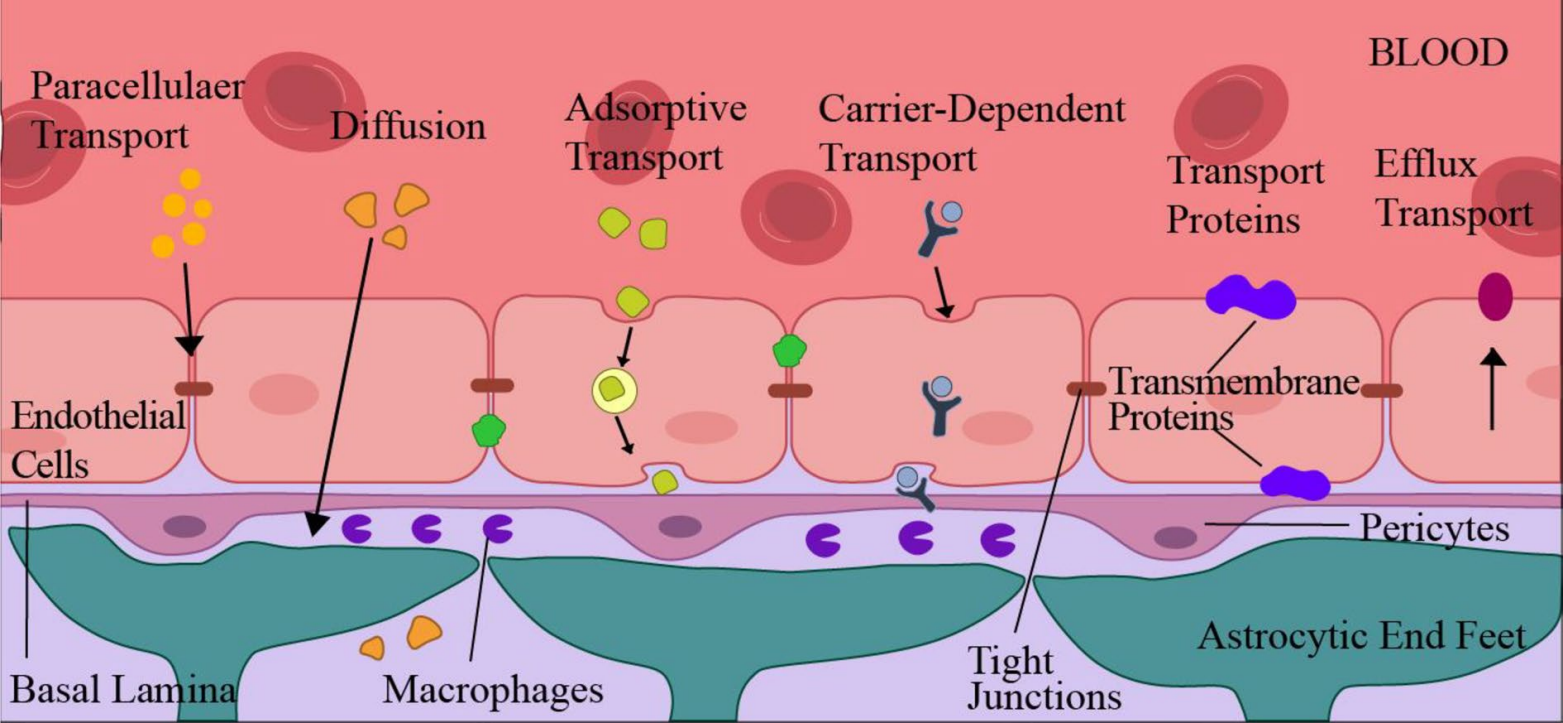
Schematic representation of methods of transport across the blood–brain barrier (i) Paracellular transport, which is obstructed by transmembrane proteins and tight junctions. The enzymes released by endothelial cells deactivate the molecules transported by paracellular means. (ii) Transcellular diffusion of hydrophobic and lipophilic molecules with the help of proteins, such as claudins. (iii) Transcytosis aided by intracellular vesicle trafficking. (iv) Carrier-mediated transcytosis with the help of transporter proteins. Transport proteins mediated transfer without making use of endocytosis and exocytosis. (vi) Multidrug resistance efflux pumps rarely undergo transport from inside to outside of the blood–brain barrier [53]

Some sections of the BBB do not possess these three layers, although they constitute most of the BBB zones. The prominent ones are the hypothalamus, Pineal gland, and area postrema, because these regions have sensory receptors, which are the hormonal regulatory regions of the brain [29]. Even after the tri-layer protection acts as a roadblock for substances entering the brain, some pathogens can still penetrate through this barrier [31]. An infection with a neurotropic virus also affects the BBB's functional homeostasis, because it changes the BBB's permeability and causes anti-inflammatory and pro-inflammatory innate immune reactions (Table 1). Numerous neurotropic viruses, such as the HIV of the Retroviridae family, the West Nile virus, the Zika virus, the Japanese encephalitis virus, the Venezuelan equine encephalitis virus of the family Togaviridae, tick-borne encephalitis virus (Flaviviridae family), and others, can enter the BBB via a haematogenous pathway and infiltrate the CNS. Viral entry into the BBB occurs via three distinct pathways: the trans-cellular pathway, the paracellular pathway, and a Trojan Horse mechanism utilising the diapedesis of infected cells [30, 31]. Researchers have found that SARS-CoV-2 can infect the respiratory system and the CNS. Following an autopsy, a recent analysis of COVID-19 patients revealed the isolation of SARS-CoV-2 from cortical neurons [32]. In addition to other organs, post-mortem tissue from the brain also contained SARS-CoV-2 RNA [32]. The data from human brain organoids has also confirmed that SARS-CoV-2 can invade neurons. The possible transport mechanisms by which any substance can move across the BBB are depicted in Fig. 3. Although the diseases penetrate the BBB, this strong line of defence does not allow neurological drugs to move across the BBB. Paracellular transport is usually impossible across the BBB due to tight junctions and the inactivating enzymes released by endothelial cells. These endothelial cells allow active and passive diffusion across the BBB through transmembrane proteins, usually claudins, which are the pore-forming structures within them [33]. Besides these hydrophobic molecules, lipophilic molecules and molecules with a weight of fewer than 500 Daltons can cross the BBB for transport to the brain through the CSF [34]. Neutral amino acids and glucose transporters transport amino acids and glucose, respectively. Monocarboxylates, glutathione, nucleosides, hexoses, amines etc., are transported with the help of facilitated diffusion, which includes transport through a transporter which induces conformational changes in the proteins present in the membrane [35]. This efflux and nutrient transportation is called vesicle-mediated transport regulation across the BBB.

**Table 1:** Neurological complications induced by various viruses following BBB invasion

Virus	Family	Neurological implications	References
SARS-CoV	Coronaviridae	Follows hematopoietic route, can enter through olfactory means and has ACE2 receptors	[28, 29]
SARS-CoV-2	Coronaviridae	High affinity binding to the ACE2 receptor that involves spike protein	[30, 31]
Lyssavirus/Rabies virus	Rhabdoviridae	Nicotinic acetylcholine present on neuronal cells act as receptor and viral glycoprotein is ligand	[40]
Japanese encephalitis virus	Flaviviridae	Increase expression of Matrix Metalloproteinase 9 which limits Reactive oxygen species	[41]
Zika Virus	Flaviviridae	Follows paracellular pathway to cross placenta, Decrease effects of Occludin and Claudin-5	[34, 35]

The third method of transport includes transcytosis; it is a mechanism to facilitate transcellular transport by enclosing the molecule to be transported in a vesicle. This vesicle penetrates through the apical membrane and transfers the material to be transported; this process is called endocytosis. The vesicle is moved across the cell (intracellular vesicle trafficking) and ejected by the basolateral plasma membrane. This process is termed exocytosis. Specific receptors are responsible for mediating transcytosis across the BBB. Some of these include albumin receptors, transferrin receptors and insulin receptors. This transcytosis mechanism operates in two ways: through adsorptive transcytosis, which functions as described previously, and the other with the help of carriers or transporters. Researchers have identified three kinds of vesicles in endothelial cells: macro-pinocytotic vesicles, clathrin-coated pits and Caveolae [36–39].

These transport mechanisms do not provide a way to transport some pivotal drugs for treating brain-related diseases. To overcome the problem of drug transport, scientists have come across other ways. The first way is transporting the drug via cerebrospinal fluid (CSF), which makes up the lining of the ventricles of the brain and subarachnoid spaces of the spine and the skull. The choroid plexus regulates drug transport via CSF, constituting the blood–CSF barrier [42, 43]. The drugs are intrathecally injected, equivalent to a slow intravenous injection. Following this, passive diffusion through the BBB occurs, which is a slow process [44]. The second method involves the use of vasoactive compounds. These compounds have the property of dilating or contracting the blood capillaries, aiding the movement of drugs. However, these are distinctive from the vasopressors and inotropes as they raise blood pressure and cardiac output. Four commonly used FDA-approved vasoactive drugs are phenyl epinephrine, norepinephrine, epinephrine, and vasopressin. The third method relies on the pharmacology of drug action in which highly lipid-soluble drugs are used to make their passage facile through the BBB via transporter proteins. These drugs include phenytoin, carbamazepine, oxprenolol, and timolol [40, 41, 45]. The fourth strategy involves interfering with endogenous transport mechanisms and blocking the efflux transporter, which pumps medicines back into the bloodstream [46]. Several other approaches have been tested for transporting drugs across the BBB. The contemporary is the nanoparticle approach in which Molecular Envelope Technology (MET) engineering is used [47]. Nanoparticles exhibit specific characteristics, such as non-toxicity, biocompatibility, small size, extended blood circulation, controlled drug release and targeted efficiency, making them suitable for drug transport across the BBB. Drug-delivery nanovesicles are designed for this purpose [48]. There have been recent experiments regarding using a polyacid (lactide–co-glycolic) for synthesising nanoparticles employed to transport drugs to treat Alzheimer’s disease. Besides this, polyethene imine and L-glutathione have been used to increase cyto-compatibility [49]. Some studies have focused on using human serum albumin-based nanoparticles and nanoparticles made up of poly ethyl cyanoacrylate to treat inflammation in brain tissue. Along with these polymeric nanoparticles, dendrimers have been used for transport across the BBB. These are unique nano polymeric molecules formed by the consecutive layering of a branching group over the other. The core molecule and the branched molecular layers are the same. There are functional groups present on the outer layer. Polyamidoaminedendrimers are being extensively used [50]. The other category of nanoparticles is micelles, amphiphilic molecules with particle sizes of 5–50 nm. They self-orient themselves with the hydrophobic portion called the tail forming the centre and the hydrophilic portion called the head directing outwards. These are usually deployed for the transport of poorly water-soluble substances. Inorganic nanoparticles such as gold nanoparticles, silica nanoparticles and carbon nanotubes are also used, but these typically lack the property of biodegradation and are hence used for bio-imaging purposes [51]. Levodopa and peptides specific to β-amyloid have been used as functional groups on gold nanoparticles in treating Alzheimer's and Parkinson's disease. Another study involved injecting mice with insulin-coated gold nanoparticles, which caused a build-up of insulin in the mice's brain, according to the results of micro-computed tomography. Carbon tubes can be multi or single-walled and consist of nano-scaled diameter graphite tubes. These possess characteristic features of functionalisation with different chemical compounds resulting in conformational, chemical, and physical changes. Drugs such as piracetam, pentoxifylline and pyridoxine have been delivered using silica nanoparticles [49]. In addition, newer discoveries about cyclic peptide nanocarriers and liposomes can be used to cross the BBB [52]. These developments highlight the need to re-evaluate some notions about drug delivery to the brain and also provide exciting possibilities for novel drug delivery techniques through various transport mechanisms.

Besides drugs, these transport mechanisms can also be apprehended to be involved in the transportation of SARS-CoV-2. Our comprehension of this relationship has improved due to numerous neurological studies showing that SARS-CoV-2 changes the BBB's functionality. Patients with minor COVID-19 infection have reported symptoms, such as dysgeusia, anosmia, and headache [30]. In addition,symptoms for patients with severe COVID-19 include delirium, disseminated encephalomyelitis, psychosis, catatonia, mania, Guillain–Barre syndrome, and seizures. Following 6-month COVID-19 infection, recent research examining the association between anxiety, sadness, and exhaustion has demonstrated that those who have long-term COVID experience serious neurological consequences [53]. This neurological evidence explains how COVID-19 could affect the nervous system via the BBB, among other mechanisms. It justifies the possibility that SARS-CoV-2 could enter the BBB through one of the above-mentioned pathways or other channels [29].

## Invasion of SARS-CoV-2 in the brain

Positive-stranded RNA makes up the genome of coronaviruses, which is encapsulated and replicates in the cytoplasm. To transmit the nucleocapsid into the host cell, the pathogen relies on the fusion of the envelope with the host cell membrane. The spike glycoprotein (S), which mediates virus entrance, must be present for a virus to enter a cell. It is designated as a class I fusion protein, and the fusion of the viral and host membranes as well as its binding to the host cell receptor are mediated by the conformational changes in the protein [54, 55]. In case of COVID-19 infection, low pH in intra-cytoplasmic vesicles is necessary for viral entrance. The entry of the virus inside the host cell is the first and the most critical stage in virus infection [57, 58]. Growth factor receptors such as the keratinocyte growth factor receptor, epidermal growth factor receptor (EGFR) [56] and transferrin receptor [57] have been used to study clathrin-dependent endocytosis. Endosomes transport endocytosed receptors to the cell surface, destroying or recycling them.

A tiny, vesicular-like structure with a coat of cytosolic protein clathrin mediates clathrin-mediated endocytosis (CME). The binding of the virion to host receptors initiates CME. An adapter protein binds the host cell's receptor due to the virion attaching to the host receptor. Due to the concentration of adaptor proteins on the inner side of the plasma membrane, when adaptor proteins bind to clathrin, clathrin can multimerize to generate clathrin-coated pits (CCP) [59]. Following the snipping of the CCP from the host membrane by membrane scission proteins Dynamin-1 (DNM1) or Dynamin-2 (DNM2), the clathrin-coated vesicle (CCV) is released [60]. This results in a bare vesicle that then transmits its viral contents into an endosome [61–63]. Viruses attached to receptors are similarly endocytosed and carried to vesicles known as early endosomes. When early endosomes mature to become late endosomes, they become more acidic (pH 6.0 to 5.5). Endosomes must be acidified for integrated viruses to establish an infection [57, 58]. The clathrin-independent pathways, on the other hand, include a caveola-dependent route. Caveolae are small, membrane-engulfed vesicles that ranging from 50 to 100 nm and are coated with caveolin-1 [2, 66, 67]. Picornaviruses [63], papillomaviruses [64], filoviruses [65], and retroviruses [66] are among the viruses that use the caveola-dependent route [66].

To enter host cells, SARS-CoV-2 must first bind to the angiotensin-converting enzyme 2 (ACE2), a receptor for the S glycoprotein [67]. The heart chambers, liver, forebrain, testis and spleen among rats and mice were revealed only to have a small amount of ACE2 mRNA, whereas a significant and comparable expression was observed in the lungs. However, both species' ileum had the highest concentrations of ACE2 mRNA [68]. This indicates that SARS-CoV-2 may use ACE2 as a cellular entry receptor. Studying the function, expression, and importance of ACE2 receptors in COVID-19 infection is crucial. SARS-CoV primarily enters target cells through clathrin-mediated endocytosis, according to recent studies employing pathogenic SARS-CoV and a SARS-CoV pseudovirus trapped in the SARS-CoV envelope. SARS-CoV entry into caveolin-1-negative HepG2 cells was significantly reduced by administration with chlorpromazine which is a clathrin-dependent endocytosis inhibitor, combined with small interfering RNA (the approach that attempts gene silencing for the clathrin heavy chain). With both the wild-type and mutated variants of the ACE2 protein transfected into the COS7 cells (cells of CV-1 origin harbouring SV40 genetic material), chlorpromazine therapy successfully blocked SARS-CoV entrance. In addition, as soon as the virus linked to ACE2, it was immediately translocated inside the virus-assistive early endosomes [69]. The S2 domain of a fusion peptide links to target cell membranes and facilitates the mixing of viral and cellular bilayers. Internally, S2 has the coronavirus fusion peptide. By interacting with ACE2, the virus makes the internal S2 subunit's S2′ location visible [70, 71] After S2′ site cleavage by transmembrane protease, which is serine 2 (TMPRSS2) at the cell surface or whether through cathepsin L within the endosomal compartment following ACE2-mediated endocytosis, the fusion peptide is released [73, 74]. The viral genome requires access to the cytoplasm, which it can only achieve while this pore grows and the cell membranes combine, making each process step crucial [72]. This shows that clathrin-mediated endocytosis may be one of the routes by which SARS-CoV-2 enters the cytoplasm.

### Neurotropism and direct infiltration of COVID-19

There has been little evidence for neurotropism in COVID-19, but the traced neurotropic properties of SARS-CoV-2 could be inferred from the neurovirulence of SARS-CoV. There is evidence that glial cells and neurons express ACE2. Axons and dendrites typically exhibit less robust ACE2 expression than the cell body of neurons. In addition, autopsy studies from SARS patients showed that SARS-CoV almost exclusively resides in neurons [73]. The neuro-invasive ability of SARS-CoV-2 must be considered a potential contributing factor for the severe respiratory symptoms in acute COVID-19 cases. In addition to symptoms connected to cranial nerves, such as hyposmia, neuroglia, hypogeusia, and hyposmia, examples of neurological symptoms seen in COVID-19 individuals include headache, vertigo, impaired consciousness, etc. Neuronal necrosis and glial cell hyperplasia were reported after a pathological investigation of the brain tissue [74]. In addition, SARS genome sequences in the neuronal cytoplasm of infected patient autopsies have been studied using light microscopy (LM), real-time polymerase chain reaction (RT-PCR) and electron microscopy (EM). All eight verified SARS autopsies revealed SARS virus particles in the neurons, and six confirmed cases of scattered red neurodegeneration and edema were documented [36]. The findings revealed that in mice engineered for human ACE2, the brain was the primary target organ for SARS-CoV [74]. Since SARS-CoV-2 has a similar sequence and mode of infection as SARS-CoV, the findings in the case of SARS-CoV are speculated to be related to SARS-CoV-2 [75].

Anti-spike or anti-nucleocapsid antibodies are used to identify cells positive to viral proteins in the medulla oblongata. SARS-CoV-2 nucleoprotein and RNA occurrence have also been reported from certain cranial nerves that emerged from the lower brainstem [14]. Evidence that SARS-CoV-2 can directly enter and get access to the brain comes from the virus' discovery in cerebrospinal fluid and brain epithelial cells. Exploring how SARS-CoV-2 penetrates the brain has been the subject of extensive research. It has been found that there are three mechanisms through which the virus can gain access to the brain [75]. The most prominent route is via the ACE2 receptors. The ACE2 gene has 18 exons and just one catalytic domain on chromosome Xp22. ACE2 balances ACE activity by catalysing the conversion of angiotensin II (Ang-II) to angiotensin 1–7 (Ang 1–7). ACE2 mainly breaks down angiotensin II into compounds that neutralize its harmful effects, including inflammation and the death of cells. Angiotensin is a hormone that leads to water and salt (sodium) intake and constricts (narrows) blood vessels to assist in regulating blood pressure. Angiotensin possesses four distinct forms, which are identified as Ang-I to Ang-IV. Angiotensin I is hydrolysed by ACE2 to produce Angiotensin 1–9, and Angiotensin II (the primary and active form of the hormone) is hydrolysed to produce Angiotensin 1–7. Antagonism exists between the Ang-II and Ang 1–7 peptides. The Ang II–AT1R axis is pro-inflammatory, profibrotic, and prothrombotic, whereas Ang II–AT2R and Ang 1–7-Masaxes are anti-fibrotic, anti-inflammatory, antithrombotic, and have vasodilatory effects. The C-terminal segment of ACE2 is a counterpart of the renal protein, collectrin that controls the movement of amino acid carriers to the cell surface, giving ACE2 various physiological roles. Due to the numerous physiological functions that ACE2 performs, SARS-CoV-2 uses it as a receptor. Both SARS-CoV andSARS-CoV-2 in complex with ACE2 have been structurally characterized. The main spike glycoprotein of SARS-CoV-2 interacts with the N-terminal of the ACE2 receptor and contains 380 amino acid substitutions that are distinct from SARS-CoV, which corresponds to variations in five of the six essential amino acids in the receptor-binding motif in between viral protein and the N-terminal region of the human ACE2. Forty-one viral spike proteins are well-recognized as significant targets for developing treatments and vaccines, because they are essential markers of host tropism. In addition, both S-protein and ACE2 contain host cell proteolytic enzymes crucial for SARS-CoV-2 entry and cell infection. To attach the S-protein and to facilitate cell entrance after receptor engagement, SARS-CoV-2 has evolved to use a variety of host proteases, notably trypsin, cathepsin L, factor X, cathepsin B, furin, elastase, and TMPRSS2. Spike protein can be activated by the host serine protease TMPRSS2 and the cysteine proteases Cathepsin B/L, opening up SARS-CoV-2 to two different entry paths for the pathogen [54, 55]. SARS-CoV-2 enters cells due to the spike protein's interaction with the extracellular domains of transmembrane ACE2 proteins and the subsequent down-regulation of surface ACE2 receptors [75].

The second route which aids SARS-CoV-2 entry into the brain is by olfactory means, as the primary route of its entry is through the nose via inhalation. The major symptoms of COVID-19 infection specified by the WHO include cough, fever, and loss of smell and taste, the prominent among these being respiratory infections. This depicts the association of SARS-CoV-2 with the respiratory tract, and the nasal passage serves as the entrance for the respiratory tract. According to studies done on transgenic mice, SARS-CoV and MERS-CoV may enter the nose and go to the brain via the olfactory nerves before fast spreading to the thalamus and brainstem [64]. Interestingly, MERS-CoV was found in the brains and not in the lungs of experimentally infected animals with low inoculum doses of the virus. This shows that the infection in the CNS predominantly caused the significant mortality rate observed in the afflicted animals. One of the main areas of the brain that MERS-CoV affects is the brainstem [76]. According to research, the novel coronavirus may attack peripheral nerve terminals before entering the CNS via a synapse-connected pathway. Researchers' studies show clathrin-coated transmission of HEV across neurons through these routes [77]. Similarly, reports of avian bronchitis virus trans-synaptic transmission have been made. In addition to bronchitis or pneumonia, intranasal avian influenza virus inoculation in mice has also resulted in brain infection. The two diseased areas of the brainstem: nucleus ambiguus and nucleus of the solitary tract, were revealed to contain viral antigens [78]. The nucleus of the solitary tract receives sensory information and respiratory tract information. In contrast, efferent fibres from the nucleus ambiguus and the nucleus of the solitary tract perfuse nasal passages, smooth muscle glands and the circulatory system, providing information about them. This explains the connection between the circulatory system and infection by the avian influenza virus.

The third possible entry route for SARS-CoV-2 is via cytokine storms. Cytokine storm happens due to excessive secretion of cytokines by the immune system. These chemicals can stimulate the functioning of other immune cells while promoting inflammation. Immunotherapy, autoimmune diseases, and infections such as COVID-19 can all result in cytokine storms. Pro-inflammatory chemicals are known to cause neuro-inflammation after viral infection. Studies are still underway to determine the link between cytokine storms and SARS-CoV-2 brain penetration [75].

### Systemic inflammation induced by SARS-CoV-2 and effect of Angiotensin II activity

Although the respiratory system is the primary site of inflammation caused due to SARS-CoV-2 infection, inflammatory response affecting extra-pulmonary tissues has also been noted in many cases globally. With a clinical phenotype that appears to be influenced by epidemiological characteristics, such as age, sex, or ethnicity, the signs and symptoms linked to this overactive immune response are highly varied and can mirror various autoimmune or inflammatory disorders. The intensity of the manifestations also varies greatly, from benign, self-limiting features to systemic disorders that might be fatal [79]. Acute respiratory distress syndrome (ARDS), a clinical syndrome characterised by acute lung inflammation and increased-permeability pulmonary oedema as a result of damage to the alveolar capillary barrier, can occur in some COVID-19 patients and result in a severe, acute virus-induced lung injury [80, 81]. Pro-inflammatory cytokine concentrations that are high, shock incidence that is enhanced, and unfavourable clinical outcomes are the hallmarks of the hyperinflammatory phenotype of ARDS. 'Cytokine storm' has become synonymous with the pathophysiology of COVID-19-induced ARDS, even though its processes are still being clarified. The terms "cytokine storm" and "cytokine release syndrome" (CRS) are widely used interchangeably to refer to immune-related dysregulation caused by the release of enormous amounts of cytokines that cause systemic inflammation, multi-organ failure, and high death rates [82]. Fever, tachycardia, tachypnoea, and hypotension are the major symptoms distinguishing CRS from systemic inflammatory response syndrome, one of the most common serious adverse effects of chimeric antigen receptor T (CAR-T) cell therapy [83]. A major modulator of the acute inflammatory response in ARDS and CRS, the pro-inflammatory cytokine IL-6 also appears to play a role in severe COVID-19 and contributes to increased C-reactive protein levels, hypercoagulation, and hyperferritinemia. However, compared to patients with CRS, ARDS, sepsis, or even influenza, patients with COVID-19 have significantly lower serum IL-6 concentrations [81].

ACE2 functions as a functional SARS-CoV-2 receptor, which causes ACE2 to be downregulated and Ang II to be expressed more prominently. The multiorgan dysfunction seen in COVID-19 patients may be explained by the fact that ACE2 is primarily expressed by vascular endothelial cells of the lung, as well as in extrapulmonary tissue, the heart, neurological system, gut, kidneys, blood vessels, and muscles on cell surfaces. Pneumonia, ARDS, sepsis, coagulopathy, a high rate of thrombosis, and organ damage are all part of the complicated clinical picture of COVID-19 patients with severe consequences. These conditions are brought on by diverse effects of highly expressed Ang II on vasculopathy, coagulopathy, and inflammation [84].

SARS-CoV-2 infection of the lungs damages the endothelium lining of the blood vessels and increases capillary permeability, allowing the virus to spread from the lungs to the pulmonary microcirculation. SARS-CoV-2 may enter the CNS after crossing the BBB by directly interacting with ACE2 receptors or by changing the tight junction proteins produced by endothelial cells lining the BBB. Proinflammatory cytokines (IL-1, IL-1, TNF-, IL-6, IL-12, etc.), thrombin, fibrinogen, and plasmin concentrations rose after SARS-CoV-2 infection and caused hypoxia that breaks down the BBB may allow SARS-CoV-2 to pass through paracellularly and enter the CNS [85]. By acting  via  a "Trojan horse mechanism" (decoy), infected leukocytes or endothelial cells can carry viruses over the blood–brain barrier (BBB) and into the brain. Viruses can also cross the blood–CSF barrier through choroid plexus epithelial cells. In wild-type mice, the S1 subunit of the spike protein is transported to the brain parenchyma through a vesicle-dependent process known as adsorptive transcytosis, even though viral RNA or particles have not consistently been demonstrated to exist in a patient's CSF or brain tissues. It is noteworthy that persons with COVID-19 neurological symptoms typically have anti-SARS-CoV-2 antibodies in their CSF. The BBB and/or blood–CSF barrier leaking are probable explanations for this [86].

### Viruses invading the central nervous system

Brain infections are less frequent than other organs and depend on unusual circumstances that enable the virus to cross the blood–brain barrier. Systemic viruses do not typically infect the brain. Those who do so might profit from infrequent occurrences such as the blood–brain barrier breaking down or an infection of immune cells that resemble Trojan horses and can cross the blood–brain barrier but release viruses. The entrance point and route may significantly impact the final symptoms produced. Neurological issues can be brought on by viruses in a variety of ways, such as apoptosis by vesicular stomatitis virus (VSV), disruption of brain cells by cytomegalovirus or additional harm brought on by the release of glutamate, DNA, and other factors that contribute to more brain damage [11]. Evidence state that viruses enter the brain via the bloodstream or, in the case of rabies, by advancing over peripheral nerves. Viruses such as rabies do not cause the death of neurons; instead, they hijack cellular transcriptional processes to activate viral instead of neuronal genes, causing the loss of neuronal function while maintaining a regular appearance on standard pathological inspection [11]. In 1918, during the influenza pandemic, caused by the H1N1 virus with avian origin genes, research revealed that those infected experienced encephalitis, lethargy, sleep abnormalities, movement disorders, and mental illnesses [87]. In addition, it has been discovered that the common herpes infection brought on by the Herpes Simplex Virus (HSV) is linked to inflammation in the brain and Alzheimer's disease (AD). HSV infection is linked to Alzheimer's due to brain inflammation, and COVID-19 can cause neurological symptoms in 36.4% of cases.

## Cognitive functioning and the effects of COVID-19

Evidence suggests that symptoms of COVID-19 persist even during the subacute and early chronic stages of the illness, lasting longer than the initial illness. Vernacular terms for "brain fog," have been associated with symptoms, such as low energy, trouble focusing, disorientation, and trouble finding the correct words. This condition is frequently called "long COVID". Various neurological effects, such as stroke, encephalopathies, inflammatory syndrome, microbleeds, and immune reactions, have been demonstrated in case studies with COVID-19 patients [88].

Cognition refers to the mental operations required for knowledge acquisition and comprehension. The different cognitive functions include thinking, knowing, recalling, judging, problem-solving etc. Higher level brain functions include planning, language, imagination, and perception. Although being the most intensively studied nervous system activities, the cellular mechanisms underlying cognitive ability in the brain still need to be discovered; one of the reasons for this may be a failure to thoroughly understand mechanisms by which non-neuronal cells in the glia may contribute to the processes of cognition. Even though electrical communication is the foundation of brain function, neurons acting alone appear to explain just a portion of complex cognitive processes, particularly those involving large temporal and spatial dimensions of integration [89].

Whether COVID-19 can induce long-term cognitive deficits or not is debatable. A correlation between human herpes virus infections and the development of dementia later in life corroborates this hypothesis [90]. Neurodegeneration may appear many years after viral infections in the CNS, as was the case with encephalitis lethargica in 1918 when extrapyramidal symptoms (uncontrolled and involuntary movement disorders) appeared long after Spanish influenza had subsided [91]. Japanese encephalitis (JE), which primarily affects children, is Asia's most frequent cause of encephalitis. Adult cases have been reported to be less frequent. The entry of the JE virus in the brain following subcutaneous inoculation is via the haematological route of infection that could be due to endothelial cell inoculation and transcellular transport in brain parenchyma as well as paracellular leakage through a compromised BBB or blood–CSF barrier. Around 45–64% of discharged patients hospitalised for the infection showed symptoms of cognitive decline [92]. Due to abrupt respiratory failure or shock from any possible causes, one-third of patients in the intensive care unit (ICU) have a severe cognitive impairment, which negatively affects their neuropsychological performance and puts them on an equal level with patients with moderate traumatic brain injury. Depression, post-traumatic stress disorders (PTSD) and anxiety are common long-term psychological implications of ICU hospitalisations. COVID ICU stays are expected to have similar effects, as evidenced by studies from various countries [93].

Researchers examined multiple brain areas to check cognitive function in 29 people who had recovered from COVID infection. They discovered that sustained attention—the ability to pay attention to vital information was compromised. The researchers hypothesized that it was linked to underlying inflammatory processes. Conversely, COVID patients are more likely to have had hypoxia or silent strokes, which can result in brain damage [94]. As previously mentioned, COVID-related strokes are widespread, specifically among the population over 70. Regularly occurring silent strokes increase the risk of dementia and major strokes. Silent strokes primarily damage the brain’s white matter, allowing easy communication between various parts of the brain. This circuit is crucial for prolonged attention and is harmed upon being damaged. A myelosuppression syndrome has been described because of COVID-19 disease. Interleukin-6 (IL-6) and various inflammatory cytokines have been reported to circulate significantly during COVID-19 infection [95]. They have a dose–response relationship with severe respiratory distress syndrome, respiratory failure, and other pathological conditions. In patients with COVID-19, autoimmune inflammatory dysregulation can contribute significantly to acute and post-acute neuropsychiatric and cognition impairments.

Depression is also a common feature of cognitive impairment. The immune system produces chemokines, pro-inflammatory cytokines, and other substances when the body contracts the COVID-19 virus. According to experts, consequences such as inflammation of the nerves, disruption of the BBB, CNS infiltration by the peripheral immune cells, induction of indoleamine 2,3-dioxygenase (IDO), dysfunction of the hypothalamic–pituitary adrenal (HPA) axis, activation of the microglia, and impaired nerve transmission, among other symptoms can occur if the body is unable to regulate these cytokines properly. These are the underlying causes of psychiatric diseases, such as depression. It suggests that even when a person recovers from COVID-19, the infection's long-term effects could lead to depression [57].

Various lines of studies revealed a correlation between immunological response and depression. This suggests that immunological decline due to COVID-19 can result in depression and other neurological problems. The several ways by which immunological loss due to SARS-CoV-2 results in neurological implications include: first, immunological manipulation due to endotoxins, interferon-alpha (IFN-α), or typhoid vaccines which leads to altered behaviour with symptoms, such as weariness, poor mood, and hypersomnia. Second, research has shown that individuals with autoimmunity, such as rheumatoid arthritis or inflammation-inducing factors, seem more likely to be depressed later in life. Third, analyses of biomarkers suggest that inflammatory markers, namely, transforming growth factors, acute-phase proteins, such as C-reactive protein (CRP), IL-6 and IL-1β, are much higher in depressed patients than healthy controls. Minimal systemic inflammation has also been proven to cause considerable pathophysiological irregularities in the endocrine system that regulates stress and anxiety, which exacerbating neuroimmune reactivity. Furthermore, research carried out in human and animal models has proved that immune defence activation might trigger brain inflammation. Higher inflammatory responses have been observed in post-mortem brain tissues of patients suffering from depression [97]. In a cognitive screening test, Montreal Cognitive Assessment (MoCA), it has been found that in individuals suffering from COVID-19 and seeking acute psychotherapy, cognitive impairment is quite prominent and is connected to a history of delirium. During inpatient rehabilitation, cognition and conscience improved dramatically, and clinically essential cognitive gains were linked to enhanced functional gains. It implies that even after recovering from COVID-19, a person may have depression due to the infection's long-term impacts [96]. The study found cognition changes post-COVID-19, emphasizing that those therapeutic interventions may be necessary for facilitating functional recovery. Despite improvement, in most samples, cognitive impairment was prevalent even after patients were discharged from inpatient rehabilitation, highlighting the significance of cognitive decline after getting infected by SARS-CoV-2.

## COVID-19 and its repercussions on the brain

**Fig. 4:**
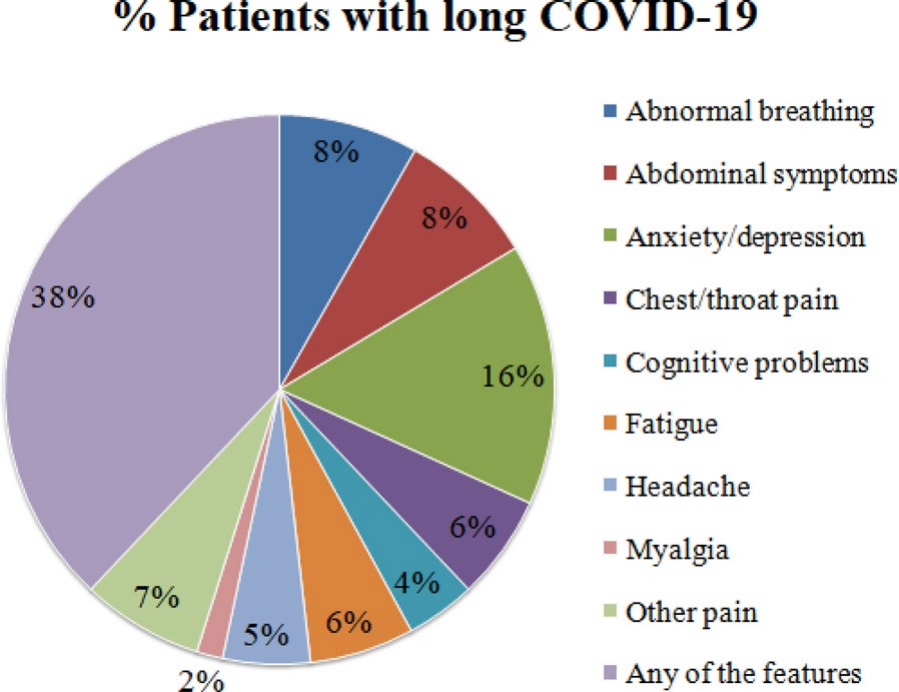
Using information from the TriNetX electronic health record network in the US, a research from the University of Oxford and also the National Institute for Health Research (NIHR) Oxford Health Biomedical Research Centre (BRC) examined long-COVID in over 2,70,000 people recovering from COVID-19 infection [103]. According to the study, nine main long-COVID symptoms were diagnosed that appear 90–180 days following the diagnosis of COVID-19 which have been depicted in the pie chart marked by different colours

Recent studies highlighting the repercussions of SARS-CoV-2 on various organs have concluded that cognitive problems such as brain fog and motor movement complications were commonly observed in individuals diagnosed with COVID-19 (Fig. 4). Major studies conclude that the virus in question can infect most human organs and tissues. Among these, the respiratory system is the most prominently affected, leading to Acute Respiratory Distress Syndrome (ARDS). In this potentially lethal condition, the lungs cannot oxygenate the body's vital organs. Aside from these, the most frequently impacted systems are the gastrointestinal, cardiovascular, and renal functions. According to a study, sustentacular cells, which support sensory neurons in the nose, are infected and damaged by the virus. These cells communicate smell and taste signals to the brain, causing anosmia and ageusia, respectively. Another distinguishable yet often overlooked impact of COVID-19 is the neurological problems patients face. Various case studies reveal the disastrous footprints of the neuroinvasion of SARS-CoV-2. These could be categorised as cerebral diseases, cerebrovascular manifestations, motor movement complications, and sensorial and cognitive symptoms.

The peripheral nervous system (PNS) has also been found to be affected by SARS-CoV-2. Infection of the vascular endothelium, invasion by circulating immune cells and penetration across the BBB or blood–nerve barrier are a few neuroinvasive mechanisms that may impact both the CNS and PNS [98]**.** Researchers looked at the medical records of 89 COVID-19 participants. Peripheral neuropathy was discovered in 3 (6.7%) of the 45 COVID-19 patients with acute respiratory problems [99]. The involvement of the PNS may be due to the deregulation of the systemic immune response caused by SARS-CoV-2. Patients with COVID-19 have been found to have secondary hemophagocytic lymphohistiocytosis, also referred to as systemic hyper-inflammation accompanying macrophage activation syndrome [100]. SARS-CoV-2 effects may produce PNS symptoms consistent with an acute polyradiculoneuropathy diagnosis even before pneumonia settles [101].

Numerous little strokes in the brain are a sign of cerebrovascular disease, escalated by atherosclerosis-narrowing of blood vessels and plaque build-up. Brain imaging can help in its detection. It often results in issues, such as memory loss, attention problems, sluggish thinking, personality changes and diseases, such as Ischemic stroke, cerebral venous thrombosis, and encephalitis [102]. Long-standing risk factors for the illness include diabetes, smoking, high cholesterol, and hypertension (chronically high blood pressure). There can be three ways in which COVID-19 can damage the brain. These include critical infection, blood clots and physiological abnormalities.

### Critical infections leading to ARDS

Because of the widespread expression of ACE2 across the brain, the virus is believed to attack the CNS. In essence, the BBB could be destroyed by SARS-interaction CoV-2's with ACE2 in the capillary endothelium, allowing the virus to reach the CNS [75]. Furthermore, there is compelling evidence that mRNA from closely related SARS viruses, such as SARS-CoV, which interacts with ACE2, is present in the brains of infected patients. Given the similarities between SARS-CoV and SARS-CoV-2, it is likely that the presence of ACE2 in brain tissues facilitates the migration of SARS-CoV-2 into the CNS and hence, causes neuro infection [69].

SARS-CoV-2 enters the brain via the olfactory nerves through the nasal canal and may infect neurons that control respiration. This information stems from a previous study that found approximately 89 per cent of SARS-CoV-2 patients needing intensive care could not breathe independently. They had neurological symptoms, such as nausea, headache, and vomiting. Almost half of the patients were ill briefly and died from respiratory failure. Although it has not been proven that neurons that control respiration are likely to be infected [104].

T-cells and antibodies are the two most crucial components of the immune system's defence against viruses. The presence of SARS-CoV-2-specific cells was observed in patients recovered from COVID-19, suggesting they may have prophylactic antiviral impact. However, some research suggests that severe COVID-19 may be brought on by an overactive immunological reaction [105]. ARDS leads to the entire lung being inflamed and developing a significant barrier of fluid that leaks into the interstitial space. In addition, these capillaries begin to leak fluid into the alveolar space, filling with proteinaceous liquid. This substance keeps oxygen from entering the bloodstream, resulting in hypoxic blood that makes breathing difficult for the patient. Viral invasion can set off a cascade of immunological reactions that can result in a cytokine storm and, death from ARDS. The cytokine storm has been found to have a significant role in the pathophysiology of SARS and MERS in clinical and animal studies [32]. In clinical investigations, critical patients had higher levels of pro-inflammatory molecules in plasma than mild to moderate patients, implying that the cytokine storm is also linked to the severity of COVID-19 [106].

### Physiological abnormalities due to low oxygen level

Brain dysfunction, such as delirium or coma found in patients with severe COVID-19, is caused by physiological anomalies of the disease, which range from high temperature to low oxygen levels to organ failures. Delirium is a well-known symptom and an indication of serious disease in older adults. Delirium is a severe insanity that can cause a changed state of consciousness, disorientation, inattention, and other symptoms of confusion and disruptions in cognition. It is common in elderly humans and is linked to adverse outcomes, such as prolonged hospitalisations and even death [107].

### Severe blood clots in blood vessels

COVID-19 can cause blood clots or bleeding in the brain, resulting in a stroke. It can destroy the protecting cells that form the BBB, allowing hazardous substances such as viruses to pass through. The condition can wreak havoc on a person's lungs to the point, where their brain runs out of oxygen [106]. According to a group of neurologists from The National Hospital for Neurology and Neurosurgery (NHNN), London and The University College of London (UCL), clinical findings of COVID-19 patients who later suffered a stroke indicate coronavirus may create clots within blood vessels (arteries) in the brain. The results recommend D-dimer testing at an earlier stage, which may lower the number of people who have more strokes or blood clots elsewhere in the body.

## Diseases caused due to neuronal invasion by SARS-CoV-2

The majority of COVID-19 cases that have been recorded primarily present with respiratory symptoms, but alarmingly, there have also been reports of cerebral issues. An examination of the literature on COVID-19's neurological manifestations and how they present in the CNS, PNS, and musculoskeletal system has been reported. In a study in which 2,36,379 patients infected by SARS-CoV-2 were subjected to a neurological examination after 6 months of getting infected, studies found that the average prevalence of psychiatric incidence was 33.62%, with a confidence rate of 95%. For patients who have undergone such a diagnosis for the first time in their lives, the prevalence rate was 12.84%. The likelihood of neurological issues in patients admitted to the Intensive Therapy Unit was 46.42%, while the estimated prevalence percentage of diagnosis of those neurological manifestations was 25.79% [108].

**Fig. 5:**
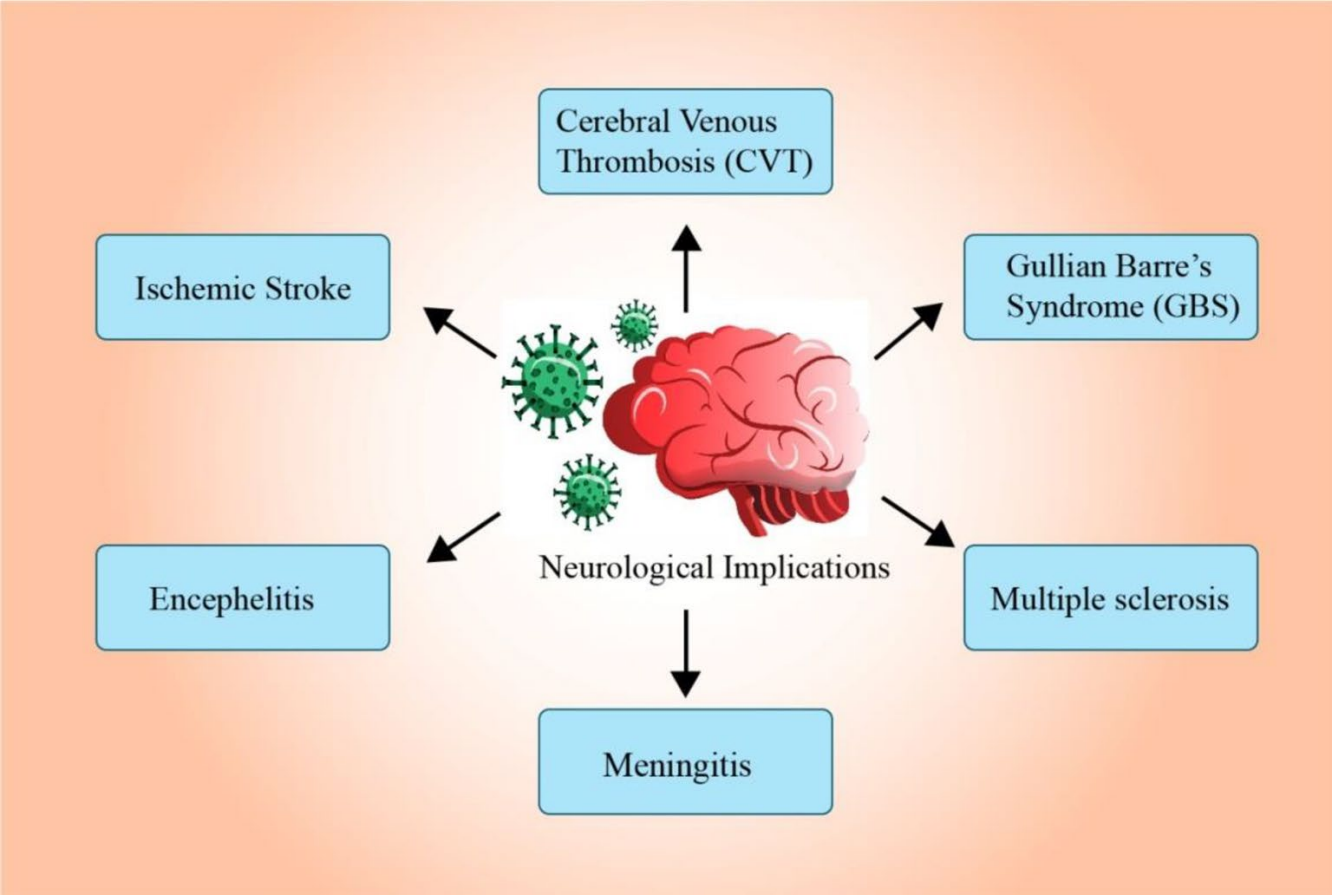
Neurological complications after COVID-19 infection [100]. Due to COVID-19 infection, disruption in the blood flow to the brain's arteries might result in one or more minor strokes. Infection in children has an increased prevalence of this form of brain inflammation (encephalitis) than adults. Guillain–Barre syndrome (GBS), sickness of the peripheral nerves and can impair motor function by reducing tendon reflexes. The insulating myelin layer that shields nerves is attacked by the immune system in the chronic illness known as multiple sclerosis (MS). MS risk is increased by disturbed immune responses brought on by an invasive virus

The early signs and manifestations of this infection include shortness of breath, fever, chills, fatigue, and severe cough, acceded by hypogeusia and hyposmia, as well as neuroinvasion by SARS-CoV-2 including severe headaches, confusion, and brain fog. Motor movement complications are also observed [87, 89]. A person infected with the virus may either suffer from all the symptoms mentioned above or not exhibit either (Fig. 5). Post-COVID MRIs (Magnetic Resonance Imaging) are advised by neurologists when patients suffer from neurological complications over a more extended period [53].

Stroke is one of the adverse consequences of COVID-19 infection, and people with cerebrovascular illnesses are more likely to develop this issue. The flowchart in Fig. 5 shows the various neurological issues that COVID-19 might cause. Among the cerebrovascular effects of COVID-19 infection are ischemic stroke, cerebral venous thrombosis, and encephalitis. Headaches, mental disorientation, and personality changes can all be caused due to blood clots in the brain's veins. Children are more commonly affected by this kind of encephalitis than adults. Guillain–Barre syndrome (GBS), a disease of the peripheral nerves and nerve roots, is frequently preceded by gastrointestinal or respiratory infection and can impair motor function by reducing tendon reflexes. There have been case reports and cohort studies of COVID-19 patients who experienced ischemic stroke, intracerebral haemorrhage, and cerebral venous sinus thrombosis. These conditions have an incidence rate of 0.5–5%. [109]. Many risk factors for cerebrovascular illnesses, such as hypertension, diabetes and smoking, also enhance the likelihood of being severely ill from COVID-19 exposure [110]. In a study conducted to investigate the correlation between COVID-19 infection and venous thromboembolism (VTE) in elderly patients with acute ischemic stroke (AIS), it was identified that the findings indicated a significantly higher occurrence of VTE among AIS patients with a history of COVID-19, irrespective of hospitalization status. The study underscores the importance of recognizing the elevated VTE risk in AIS patients with a prior COVID-19 history and implementing appropriate interventions for improved clinical outcomes [111].

Studies indicate that SARS can induce orchitis and affect the brain and respiratory system. In the study, all infected testes had thicker basement membranes, leukocyte infiltration, widespread death of germ cells, and few or no spermatozoa in the seminiferous tubule [112]. Although the pathogenesis, has not yet been fully explored, it is thought that the same mechanisms that cause the disease in the primary target organs also affect the skin. According to systematic reviews, dermatological signs can occur at a frequency of 0.2–45% or close to 6% [113].

Although the precise mechanisms of the SARS-CoV-2 infection-induced cardiac damage are unknown, SARS-CoV-2 is thought to have two potential effects on the heart. First, ACE2 receptors, abundantly expressed in the lungs and cardiovascular system, may allow the virus to penetrate the cardiac tissue and harm it directly. Second, the virus may be causing an uncontrolled cytokine storm that harms the cardiovascular and other organ systems. SARS-CoV-2 also has a pathogenic mechanism in the colon, which is supported by detecting SARS-CoV2 RNA in human faeces. The abundance of the ACE2 receptor, found in the ileum and colon, is thought to have a role in how SARS-CoV-2 affects the digestive system. ACE2 is highly expressed in the human small intestine. As a result, foreign pathogens and food particles are directly exposed to enterocytes. The normal intestinal flora is hypothesised to be disrupted by SARS-CoV2 viral attachment to the ACE2 receptors of the digestive tract, which causes a variety of GI symptoms, most notably diarrhoea.

### Ischemic stroke

A stroke happens when the body's flow of blood flow to the brain is interrupted. Ischemic stroke, also known as brain or cerebral ischemia, is the most common type of stroke and occurs due to a blockage in the arteries surrounding the brain. The obstruction causes the brain to get less blood and oxygen, which damages or kills brain cells and may become irreparable if blood flow is not quickly restored. ACE2 hydrolyzes Ang-II into angiotensin-(1–7), and the ACE2/angiotensin-(1–7)/MAS counteracts the unfavourable effects of the RAS while also having anti-inflammatory actions, in addition to serving as the receptor for SARS-CoV and SARS-CoV-2. SARS-CoV-2 infection has been shown to downregulate ACE2 expression in cells and cause severe organ damage, disrupting the physiological equilibrium between ACE/ACE2 and Ang-II/angiotensin-(1–7) [93]. In conjunction with this, without angiotensin-17, inflammation may increase Von Willebrand's factor (VWF), which further couples with factor VIII, sometimes referred to as an anti-haemophilic factor and is a crucial blood-clotting protein ultimately causing arterial thrombosis. The most common type of ischemic stroke seen in patients with COVID-19 is Embolic stroke. It happens when the blood clots break off from one area of the blood vessel and block an artery or tiny capillaries, causing a high inflammatory response leading to hyper-coagulation. Elevated D-dimer levels have been observed, indicating prominent blood clots in neurological patients [114]. The age group most commonly affected by ischemic stroke are the young and middle-aged patients (30–45 years). Strokes are one of the major cardiovascular disorders that affect the brain and results in inefficient blood supply to the brain [115]. Strokes can be categorized as haemorrhage or Ischemic. The proper causation of strokes remains unclear for different cases but specific infections (bacterial or viral) lead to the formation of strokes. Different medical records showed a clear relation between stroke cases and COVID-19 infection, which increased the risk of ischemic and cryptogenic stroke in general [116]. In the case of children, the relation was infrequent, and there was no evidence between these two conditions [117]. However, to drive any conclusion regarding the relation between strokes and SARS-CoV-2, testing must be increased, followed by data analysis and further interpretation. Another unusual finding was that these patients had no significant risk factors, such as high cholesterol levels, diabetes, or high blood pressure problems [118].

The COVID-19 epidemic has had a profound impact on stroke care globally. During this time, the Society of Vascular and Interventional Neurology supplied advice on treating stroke. Stroke risk has gone up due to COVID-19, and healthcare delivery has been made more difficult. Hospitals have prioritised operations such as thrombectomy to assess and treat stroke patients to solve these issues quickly. The need to raise public knowledge about stroke has been emphasised. Patients with COVID-19 have been isolated from others to stop the spread of the disease. Stroke care quality has been maintained through multidisciplinary teamwork and procedural optimisation. With the help of these experiences, future pandemic scenarios can be prevented, and stroke care can be provided effectively worldwide [119, 120].

### Cerebral venous thrombosis

**Fig. 6:**
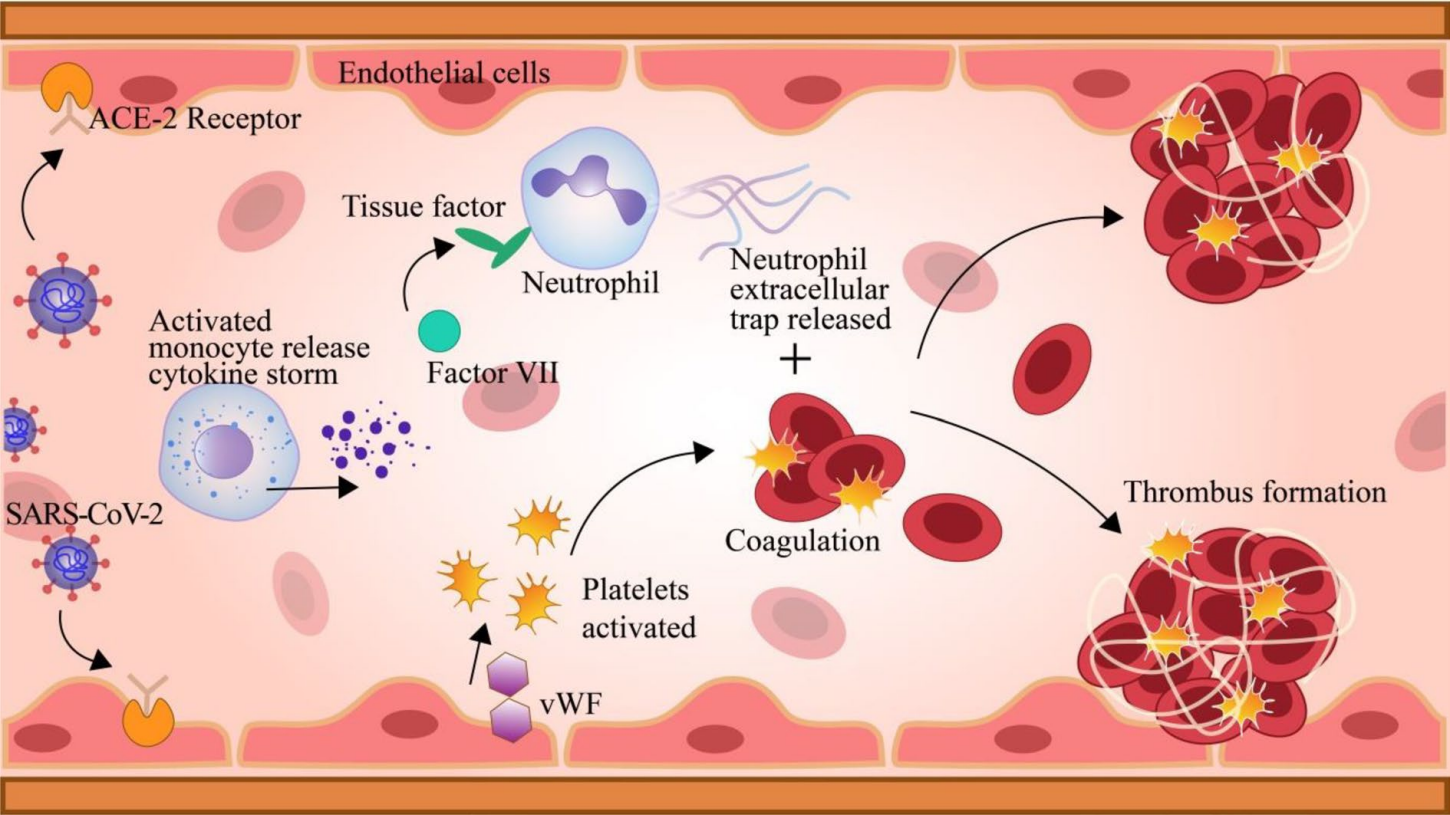
SARS-CoV-2 infects the host through binding to ACE2 receptors on endothelial cells, causing systemic and vascular inflammation, as well as monocyte activation and the release of pro-inflammatory cytokine storm. Neutrophil recruitment and tissue factor (TF) activation occur as a result. Continuous recruitment of neutrophils releases the Neutrophil Extracellular Trap (NET), which promotes thrombosis. The TF subsequently attaches to the coagulation factor VII (FVII), which activates the coagulation process. vWF (von Willebrandfactor) is released, and platelets are activated, which further accelerate coagulation [93, 98]

Cerebral Venous Sinus Thrombosis (CVST) is a condition leading to the formation of blood clots/thrombus due to blockage of a vein in the brain (Fig. 6). This causes the blood flow to cease and build up pressure, causing severe headaches and may also damage the surrounding tissues. This may result in various stoke symptoms, such as confusion, loss of movement and consciousness, blurred vision, seizures, and coma. Many researchers concluded that this disease is based upon multi-factorial symptoms and results due to pro-thrombotic risk factors in synergy with clinical conditions, such as infections [121].

A recent study reveals that thrombotic events and severe thrombocytopenia have been linked to immunisation against SARS-CoV-2. With both ischemic and hemorrhagic consequences, this syndrome, known as VITT (vaccine-induced immune thrombotic thrombocytopenia), has been linked to a high risk of catastrophic outcomes. It is still necessary to determine the best course of action. Two patients with VITT-associated CSVT were treated with high-dose intravenous immunoglobulins (IVIGs), corticosteroids, and argatroban in the hyperacute phase, followed by dabigatran, with outstanding results, according to the study [19].

### Guillain Barre’s Syndrome

**Fig. 7:**
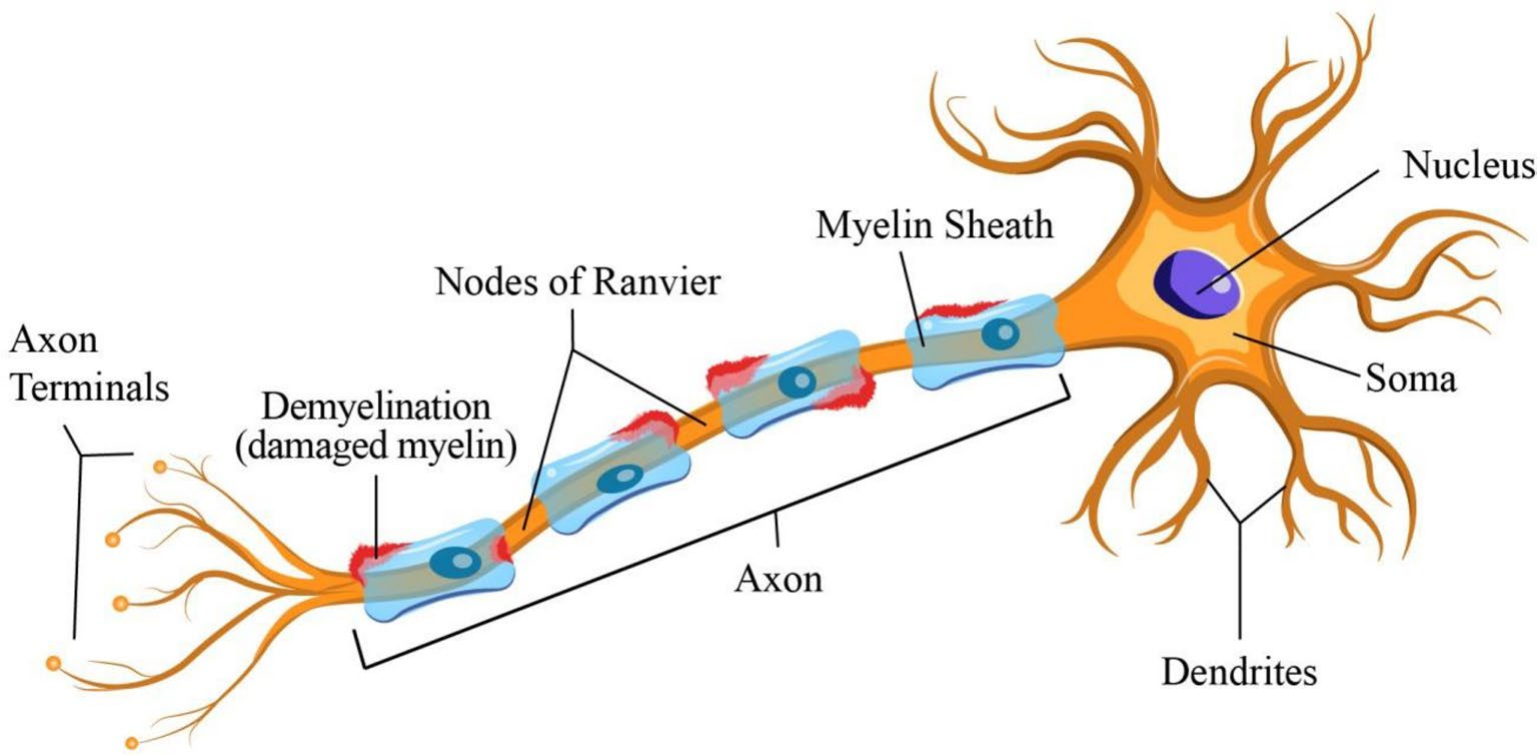
Process of demyelination in neurons resulting in inefficient transmission of nerve impulses. Following an infection, GBS is a severe immune-mediated neurodegeneration. It is induced by the autoimmune damage to nerves in the PNS. An infection triggers this immune response, and since specific chemicals on infectious bacteria and viruses mirror those on nerve cells, this makes them targets of attack

Guillain Barre’s Syndrome, or GBS, is when a myelinated neuron is damaged and loses its ability to transmit the nerve impulse properly. During viral or bacterial infection, the human body produces an immune response that corresponding to activity against viral or bacterial cells [123]. In some cases, the immune system acts against its neural system, damaging a neuron’s myelin sheath. This affects the neuron’s functioning and proper transmission of nerve impulses [124]. Demyelination of neurons and improper signal transmission is depicted in Fig. 7.

Since the pandemic, reports have confirmed increased GBS cases following COVID-19. The International GBS Outcome Study (or IGOS), a global cohort of GBS patients, was used by researchers to study patients from January 30 through May 30 2020. During this time, the study included 49 GBS patients from different countries. This cohort analysis reported that COVID-19 infection was present in 22% of the GBS patients. All of these patients were above 50, and the majority (65%) had a demyelination form of GBS and facial palsy. 73% of the COVID-19-infected Guillain–Barré patients had elevated inflammatory markers at hospital admission [124].

### Multiple Sclerosis (MS)

A demyelinating illness of the nerve cells, multiple sclerosis (MS) is a long-lasting condition, where the immune system destroys the myelin sheath that insulates [125]. The virus's disruption of immune responses increased the risk of MS and was reported in numerous people post-COVID-19 infection [126]. The causal agent of MS in humans is not well-known, but there are many theories. Some suggest Vitamin D deficiency or inadequate consumption of Vitamin D could lead to MS [127]. At the same time theories state that the effect of viruses on the neural system contributes to this condition, for instance, in the case of Epstein Barr Virus (EBV) infection [128]. Elevated levels of antibodies against EBV antigens before the onset of MS are good indicators of the relation of EBV with MS [69, 103].

Multiple factors contribute to the pathophysiology of MS, and compelling data indicate a probable viral trigger-like EBV. The olfactory bulb is the direct entry point for SARS-CoV-2 into the CNS, where it can lead to neuronal injury and prevent oligodendrocytes (OLs) differentiation and remyelination. Microglial cell death, which results in poor clearance of myelin debris, and the release of neurotoxic soluble substances from activated astrocytes are two processes that contribute to these pathologic reactions. There are two ways that SARS-CoV-2 infection could aggravate MS. First, as a direct result of a pro-inflammatory reaction, it may cause MS relapses in affected patients. Second, it may permanently change the cellular and structural makeup of the CNS in infected individuals, raising their long-term risk of developing MS. Limited retrospective studies have been done until now to investigate the aggravation of MS symptoms after SARS-CoV-2 infection, and those that have been found have yielded conflicting results. To demonstrate a rise in the disease's incidence or its rate of recurrence during COVID-19, extensive epidemiological research would be required [27].

### Meningitis

**Fig. 8:**
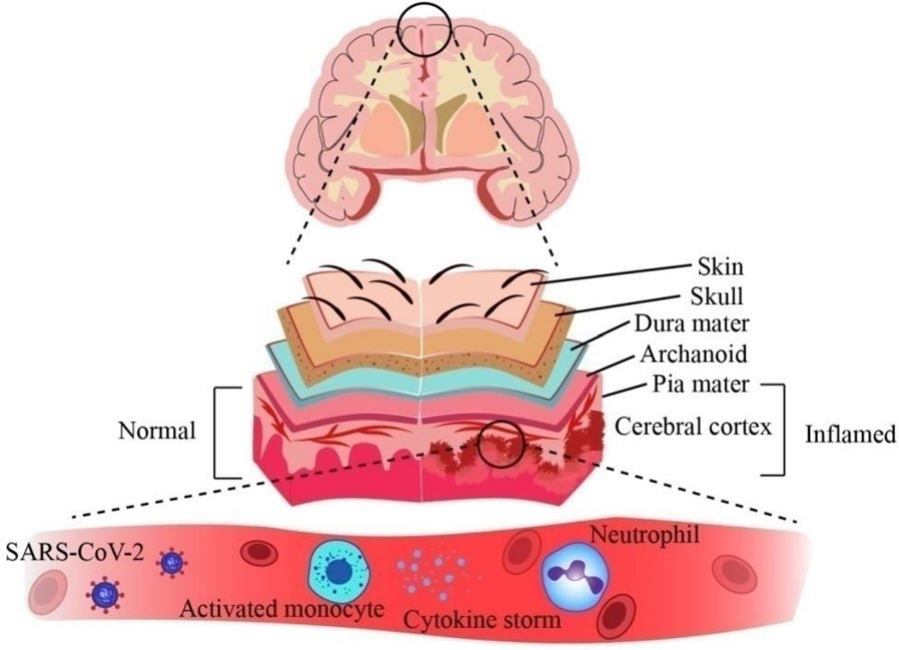
Inflammation of meninges leading to meningitis. In addition to retrograde propagation from nerve endings and bloodstream transmission, viruses can also reactivate from a dormant condition inside the nervous system and go to the meninges, composed of three layers, resulting in an inflammatory reaction brought on by a virus as it progresses through the subarachnoid space and reaches the CNS. The prognosis is worse for encephalitis, which happens when the brain parenchyma is inflamed

Meningitis is a viral infection which results in the inflammation of the meninges of the brain and spinal cord [129]. Some clinical signs and symptoms of meningitis include fever, vomiting, and headaches. Meninges are the membranous layers that protect the brain and spinal cord. Usually, bacterial or viral infections take place before meningitis. Traces of SARS-CoV-2 have been found in the CSF, which led to the development of meningitis and encephalitis in a patient [13]. Inflammation resulting from meningitis is shown in Fig. 8. In some cases, RBCs have been reported in CSF, pointing to a BBB breach, which can happen in SARS-CoV-2 infection and has been associated with cytokine storm disorder. Furthermore, the observation that individuals with severe SARS-CoV-2 infection respond to IL-6 receptor blockers supports the theory that COVID-19 patients with cytokine storm syndrome incur severe symptoms and brain damage [130]. Along with CT (Computed Tomography) scans, brain MRI scans must be done for detailed imaging to diagnose inflammation in the brain.

### Encephalitis

Encephalitis is similar to meningitis. Meningitis leads to inflammation of the meninges, while in the case of encephalitis, the entire brain is inflamed [106, 107, 131]. However, solid conclusions cannot be derived from data generated in low diagnostic studies and testing levels.

Incidences of encephalitis in patients admitted to ICUs for respiratory distress, and cases of SARS-CoV-2 infections that mainly target the CNS have been reported [132]. The SARS-CoV-2 infection was linked to every occurrence of encephalitis in this prospective multicentre research which included data from 13 neurological facilities in Italy. The cerebrospinal fluid (CSF) analyses, common electroencephalograms, and brain MRI were all performed on each of the 25 individuals. The findings show that SARS-CoV-2 infection is related to a range of encephalitis types, including limbic encephalitis, tuberculosis-related meningo-encephalitis, acute necrotizing encephalitis (ANE)/acute demyelinating encephalomyelitis (ADEM) with varying clinical presentations, therapeutic responses, and prognoses. The incidence of encephalitis cases was determined to be 50 per 100,000 as per the study's findings [133].

## Probable cerebrovascular disease pathways

Concerns about direct vascular damage resulting from SARS-CoV-2 infection are raised for patients with COVID-19 who have macro and microvascular thrombosis in both arterial and venous circulations. D-dimer is protein fragment produced upon dissolution of blood clot in the body. D-dimer is typically only detectable at very low levels or not at all unless the body is creating and dissolving blood clots. The blood's D-dimer level may then significantly increase. Systemic hypercoagulability indicated by elevated D-dimer levels that help to identify presence of blood clots, disordered immune responses, direct cytotoxic effect on the CNS, virus uptake by ACE2 receptors, cardiorespiratory distress causing hypoxemia have all been proposed as contributing factors to increased ischemic strokes [134].

### Endothelial cells, ACE-2 receptors and SARS-CoV-2

Compared to the S protein of SARS-CoV, the S protein of SARS-CoV-2 exhibits a greater affinity for ACE2. The peptidase ACE2 is found on the surfaces of immune cells, neurons, vascular endothelium, smooth muscle, and alveolar and gastrointestinal epithelial cells. This suggests that SARS-CoV-2 may target vascular endothelial cells mainly via the ACE2 receptor, leading to endothelial cell dysfunction and endothelial barrier breakdown. When the endothelium is damaged, exposed sub-intimal collagen can activate and assemble platelets, leading to thrombosis and cerebral ischemia [135]. ACE2 plays a role in the neural regulation of numerous physiological processes, including stress response, neurogenesis, metabolism, and cardiovascular activity, due to its extensive distribution in the brain. It has been observed that SARS-CoV-2 spreads through the neurons to other parts of the mouse model's brain after entering the olfactory bulb. Numerous COVID-19 patients have reported olfactory and gustatory dysfunctions, pointing to the olfactory bulb's role in SARS-CoV-2 infection.

### Cardiovascular and cerebrovascular implications of COVID-19

COVID-19 patients often display elevated D-dimer levels, indicating a hypercoagulable state with active coagulation and fibrinolysis systems [135, 136]. Inflammatory factors, elevated plasma levels of tissue factors, and platelet aggregation may be stimulated by greater sympathetic activity and more extensive micro-endothelial damage, which alter capillary blood flow and cause blood coagulation [109, 110]. This heightened procoagulant state poses a significant risk of blood clot formation, potentially leading to cerebrovascular complications, such as ischemic strokes. The presence of thrombotic events, including deep vein thrombosis (DVT), pulmonary embolism (PE), and venous thromboembolism (VTE), highlights the potential for blood clots to form and travel to various parts of the body, including the brain. Severe inflammation, diffuse intravascular coagulation (DIC), immobilization, and hypoxia associated with lung injury in COVID-19 patients further contribute to the increased risk of thrombotic events [137]. Thus, healthcare professionals must closely monitor and manage cerebrovascular implications in COVID-19 patients, especially those with pre-existing cardiovascular conditions, to optimize patient outcomes and prevent potential adverse effects on cerebrovascular health.

In the context of COVID-19, the elevated D-dimer levels and hypercoagulable state pose significant cerebrovascular implications, particularly when considering the impact of changes in oxygen concentration on the brain. Hypoxia, caused by alterations in oxygen levels, can lead to pathophysiological changes that directly affect the brain. The potential harm to vital organs, including the brain, is a serious concern if hypoxia is not promptly addressed. In the brain, hypoxia can raise cerebral capillary pressure and intracranial blood flow, leading to tissue fluid generation. In addition, hypoxia causes membrane lipid peroxidation and the release of endogenous inhibitors. Anaerobic glycolysis produces elevated levels of oxygen free radicals, lactic acid, and lipid peroxides, which compromise the antioxidant system and lead to blood–brain barrier (BBB) dysfunction [138]. When combined with the procoagulant state indicated by elevated D-dimer levels, the risk of cerebrovascular complications, such as ischemic strokes, becomes even more pronounced.

Acute infection, as seen in COVID-19, can activate self-protective defenses in the human body, leading to potential stress responses. This can result in the release of hyperglycemic hormones, exacerbating insulin resistance and causing elevated blood glucose levels [139]. Hyperglycemia, in turn, may contribute to increased oxidative stress by promoting the overproduction of reactive oxygen species (ROS) in the mitochondrial electron transport chain. These ROS can further activate various events associated with the progression of atherosclerosis, including pathways, such as the protein kinase C pathway and the generation of advanced glycation end-products [139].

According to research findings, stress has been identified as an independent cause of stroke, indicating its potential impact on cerebrovascular health. However, the precise link between cardiovascular diseases and acute psychological stress remains uncertain. Previous studies have demonstrated that under conditions of pathophysiologic stress, the production and release of plasminogen activator inhibitor type-1 (PAI-1) increase. PAI-1 is a serine protease inhibitor that specifically interacts with tissue plasminogen activator (t-PA). This interaction leads to the rapid inactivation of t-PA, and PAI-1 may play a significant role as a key mediator in stress-induced hypercoagulability and thrombosis [140].

Furthermore, SARS-CoV-2 has been found to infect the pancreas via the ACE2 receptor, which is more abundantly expressed in this organ. Consequently, this viral infection can lead to pancreatic damage and impaired insulin production, resulting in the emergence of hyperglycemia, even in individuals without pre-existing Diabetes Mellitus (DM). In addition, individuals with pre-existing DM may experience worsened symptoms if SARS-CoV-2-induced damage affects their pancreas. Peripheral insulin resistance is also induced by the inflammatory response and cytokine storm triggered by COVID-19, characterized by significant increases in the levels of inflammatory markers, such as tumor necrosis factor-alpha (TNF-alpha) and interleukin-6 (IL-6) [141]. Various disorders and complications associated with COVID-19 are described in Table 2.

**Table 2:** Various neurological disorders and risk factors associated with COVID-19

Symptoms	Area affected	Risk factors	References
Ischemic stroke	CNS	▪ Atrial fibrillation ▪ Elevated blood pressure ▪ High blood cholesterol ▪ Diabetes ▪ Extra cranial carotid disease ▪ Intracranial atherosclerotic illness	[115]
Cerebral Venous Sinus Thrombosis (CVST) or Cerebral Venous Thrombosis (CST)	CNS	▪ Obesity ▪ Thyroid disease ▪ Pregnancy ▪ Oral contraceptives ▪ Genetic thrombophilia ▪ Anaemia	[142]
Gillian Barre’s Syndrome (GBS)	PNS	▪ Older age ▪ Acute muscle weakness	[143]
Multiple sclerosis	Spinal cord	▪ Older age ▪ Chronic ailments	[144]
Chemosensory dysfunction	PNS	▪ Chronic nasal & sinus disease	[145]
Skeletal muscle damage	Musculoskeletal system	▪ Diabetes ▪ Hypertension	[146]
Encephalitis/meningitis	CNS	▪ Delirium ▪ Psychosis	[144]
Intracerebral haemorrhage	CNS	▪ Hypertension ▪ Raised inflammatory markers	[145]

## Diagnosis and prognosis of neurological implications

Among the people diagnosed with COVID-19, many patients developed various neurological disorders. The spectrum of diseases ranged from Post-traumatic stress disorder (PTSD) to Guillain–Barre’s Syndrome. The interaction of SARS-CoV-2 with the brain cells of the human body and its ability to interfere with the functioning of cells suggested the virus’s scope to alter brain cell activity leading to further disorders.

SARS-CoV-2 can be detected by two major methods. First is the molecular biology-based RT-PCR technique, and the other is immunological testing that detects specific IgG and IgM antibodies. Neurological implications appear during the first 2 weeks after the beginning of infection symptoms. RT-PCR assays of CSF may detect negative results for COVID-19 infection as RNA levels in CSF are low during the initial stages. Moreover, all neurological complications are not necessarily associated with direct viral invasion. These implications can be triggered via systemic viral infections, such as neurological autoimmune diseases, para and post-infectious nervous system autoimmune diseases and cerebrovascular and metabolic syndromes. When RT-PCR tests lead to negative results specific IgM antibodies are detected in CSF against COVID-19. Hence, this biomarker may be helpful in the detection of SARS-CoV-2 neuroinfection [146].

The presence of the virus should be analysed by examining the inflammatory profile that includes pleocytosis (increased cell count), dysfunctioning of the blood–CSF barrier, high content of protein and intrathecal generation of immunoglobulins as well as through albuminocytological dissociation, which include a high level of protein and normal cell count in CSF [146].

The prognosis of the effects of SARS-CoV-2 on CNS depends on various factors, such as age, comorbidities, immunocompromised status and disease severity. As this virus enforces its mechanism via the activation of the inflammatory response, hypercoagulability, and reduced anti-coagulability processes, inflammatory biomarkers and coagulation examinations may be predictors of prognosis [119, 120]. Liu et al. provided details on biomarkers which are the predictors of prognosis. Fibrin degradation products (FDP), Prothrombin time (PT) and D-dimer are predictive biomarkers for increased mortality, and antithrombin III (ATIII) is a prognostic biomarker for increased survival of ICU patients, according to a coagulation parameter analysis of samples taken from COVID-19-affected ICU patients [146].

Inflammatory markers, such as CRP protein and IL-6, may also predict severity of this disease [119, 121]. A study by Liu et al*.* of 140 COVID-19 patients reports a 67.9% and 65% rise in IL-6 and CRP protein levels in serum, which corresponding to the severity of the disease. Increased levels of these inflammatory markers characterize a more severe disease state, that is, more significant than 32.1 mg/mL and 48.1 mg/L, respectively. Hence, both these markers are valuable for the prognosis of this disease [147].

Acute stroke instances and issues with salt and water balance have both been significantly impacted by the COVID-19 pandemic. Particularly in elderly patients with pre-existing cardiovascular and cerebrovascular risk factors, COVID-19 raises the risk of acute ischemic strokes [148]. Salt balance abnormalities, particularly hyponatremia, are frequently experienced by COVID-19 patients. Patients with acute stroke with salt and water imbalances tend to have more severe conditions and a higher mortality risk. The fundamental source of these imbalances will determine management solutions, which should be modified accordingly. Customised strategies based on the particular aetiology are required [148]. Neurological implications result from hypercoagulability in conditions, such as cerebrovascular diseases, haemorrhagic stroke and ischemic stroke [149]. These can be managed timely to increase survival, resulting in a favourable prognosis [119, 122]. Ghannam *et al*. reported that COVID-19 leads to the activation of the platelets and the clotting cascade [119, 122]. Therefore, regarding hypercoagulability, the prognosis depends highly on the timely administration of thrombolytic therapy and should be viewed favourably. Decrease in the number of lymphocytes and platelet count, remarkable increase in neutrophils to lymphocytes ratio (NLR), D-dimer, PT, aspartate aminotransferase (AST), alanine transaminase (ALT) and Lactate dehydrogenase (LDH) highlight poor prognosis that probably results due to increased severity of the disease and hence, mortality [119, 123, 150].

To tackle the ongoing global epidemic, it is imperative to repurpose medications that may enter human trials swiftly. Strong virtual solutions such as repurposing are emerging as a way to address challenging situations, among the five FDA-approved repurposing medications that are already in use to treat a variety of disorders. Umifenovir and Pregnenolone may have potential inhibitory effects on the majority of SARS-CoV-2 proteins, according to docking research [151].

## Conclusions and future directions

COVID-19 caused due to SARS-CoV-2, was declared a global pandemic in 2020. As per the WHO epidemiological data, more than 650 million people have been infected with the virus since then. Around 6.6 million people have died from this disease worldwide. Numerous studies have indicated the ability of SARS-CoV-2 to invade the nervous system and disrupt the normal functioning of the human brain. The ability of the pathogen (SARS-CoV-2) to capture the host endocytosis system ensures the logarithmic replication of viral components; it usually takes place at low pH. This process is preceded by the interaction of the virus with ACE2 receptors on the host cell. SARS-CoV-2 exhibits neurotropic properties which can be inferred from its neuro-virulence. Studies on animal models revealed the ACE2 receptor interaction with SARS-CoV-2 and the viral entry through respiration that further spread in different parts of the brain, showed that the virus can directly enter the brain and disrupt its functioning via replication of the viral genome. The mechanisms of SARS-CoV-2 entry into host cells involve the spike (S) protein binding to its receptor, angiotensin-converting enzyme 2 (ACE2), and subsequent membrane fusion. Cytokine storms that occurred during the infection can promote inflammation inside the body and it could be another way for virus infiltration of the human brain. The infiltration of SARS-CoV-2 can lead to different neural complications, such as cerebrovascular manifestations, motor movement complications and cognitive decline. ARDS resulting from COVID-19 infection is the major cause of death in patients as it affects oxygen levels and lead to wide variety of abnormal physiological conditions, blood clots and organ failures. The manifestation of SARS-CoV-2 in brain can be recognized by acceded hypogeusia and hyposmia. The presence of blood clots in the brain can result in mental disorientation and chronic headaches.

Major cerebrovascular symptoms and diseases that follow COVID-19 infection include ischemic strokes, cerebral venous thrombosis, Guillain–Barre’s Syndrome (GBS), Multiple Sclerosis (MS), meningitis and encephalitis. In addition, lack of oxygen supply to the brain during the viral infection can result in different pathophysiological changes, such as hypoxia. Apart from cerebrovascular symptoms, psychological stress during cerebrovascular disorders could be a key mediator in stress induced thrombosis. The presence of SARS-CoV-2 in brain tissues after autopsies and occurrence of cerebrovascular disorders in patients recovering from COVID-19 infection indicate neuropathogenecity of SARS-CoV-2 and its ability to integrate with the neural system, disruption of cognitive functioning and promoting variety of neurological disorders.

## Data Availability

Data sharing is not applicable to this article as no datasets were generated or analyzed during the current study.

## Abbreviations

ACE: Angiotensin converting enzyme
ARDS: Acute respiratory distress syndrome
BBB: Blood–brain barrier
CNS: Central nervous system
CSF: Cerebro spinal fluid
CVST: Cerebro venous sinus thrombosis
GBS: Guillain Barre’s syndrome
MERS: Middle Eastern respiratory syndrome
MS: Multiple sclerosis
NETs: Neutrophil extracellular traps
PNS: Peripheral nervous system
SARS: Severe acute respiratory syndrome
TMPRSS: Transmembrane protease serine
vWF: Von Willebrand factor
